# Epibiotic fauna of the Antarctic minke whale as a reliable indicator of seasonal movements

**DOI:** 10.1038/s41598-022-25929-1

**Published:** 2022-12-23

**Authors:** S. Ten, K. Konishi, J. A. Raga, L. A. Pastene, F. J. Aznar

**Affiliations:** 1 Marine Zoology Unit, Cavanilles Institute of Biodiversity and Evolutionary Biology, University of Valencia, Paterna, Valencia Spain; 2 Institute of Cetacean Research, Tokyo, Japan; 3 Centro de Estudios del Cuaternario de Fuego-Patagonia Y Antártica, Punta Arenas, Chile

**Keywords:** Ecology, Zoology

## Abstract

Antarctic minke whales, *Balaenoptera bonaerensis*, breed in tropical and temperate waters of the Southern Hemisphere in winter and feed in Antarctic grounds in the austral summer. These seasonal migrations could be less defined than those of other whale species, but the evidence is scanty. We quantitatively describe the epibiotic fauna of Antarctic minke whales and explore its potential to trace migrations. Seven species were found on 125 out of 333 examined Antarctic minke whales captured during the last Antarctic NEWREP-A expedition in the Southern Ocean: the amphipod *Balaenocyamus balaenopterae* (prevalence = 22.2%), the copepod *Pennella balaenoptera* (0.6%); three coronulid, obligate barnacles, *Xenobalanus globicipitis* (11.1%), *Coronula reginae* (8.7%), *C. diadema* (0.9%); and two lepadid, facultative barnacles, *Conchoderma auritum* (9.0%) and *C. virgatum* (0.3%). Species with prevalence > 8% exhibited a modest increase in their probability of occurrence with whale body length. Data indicated positive associations between coronulid barnacles and no apparent recruitment in Antarctic waters*.* All specimens of *X. globicipitis* were dead, showing progressive degradation throughout the sampling period, and a geographic analysis indicated a marked drop of occurrence where the minimum sea surface temperature is < 12 °C. Thus, field detection -with non-lethal methodologies, such as drones- of coronulid barnacles, especially *X. globicipitis*, on whales in the Southern Ocean could evince seasonal migration. Future investigations on geographical distribution, growth rate, and degradation (for *X. globicipitis*) could also assist in timing whales’ migration.

## Introduction

In 1998, two species of minke whale were recognized based on the review of the morphological and genetic information: the Antarctic minke whale, *Balaenoptera bonaerensis* Burmeister, 1867, and the common minke whale, *B. acutorostrata* Lacépède, 1804^1^. Antarctic minke whales are largely restricted to the Southern Hemisphere, where they are sympatric with a dwarf form of common minke whale^2,3^, but Antarctic minke whales are relatively more frequent south of 65°S^4^. The abundance of Antarctic minke whales was estimated at 515,000 individuals (95% CI 361,000–733,000) based on sighting data collected in the period 1992/93–2003/04^5^.

Knowledge of distribution and migration patterns of the Antarctic minke whale is very limited particularly in mid and low-latitude waters, due to little research effort, low detectability at sea, and risk of confusion with the dwarf common minke whale^3,4,6–10^. It seems that Antarctic minke whales breed in sub-tropical waters of the South Atlantic, South Pacific, and Indian Oceans during winter and migrate southwards to feed on Antarctic krill during the austral summer^7,11–13^. However, direct evidence for this link is limited. Firstly, there are observations of Antarctic minke whales in the Southern Ocean all year round^14–16^, and at lower latitudes during the austral summer (e.g. Brazil, South Africa, Uruguay)^10,17,18^. Secondly, the arrival time to putative wintering and feeding grounds is variable and seems to depend on age and reproductive condition^19^. Thirdly, the extent to which Antarctic minke whales wintering in different ocean basins mix in the Southern Ocean is unclear, although available evidence suggests that populations are segregated into at least two stocks in the Indo-Pacific sector of the Antarctic^11,20^. Thus, the acquisition of further data on movement patterns is considered a priority for the development of conservation policies for this species^4^.

Since the beginning of the past century, epibionts have been used as useful ecological markers of diverse aspects of cetacean biology, including movements and stock structure [see e.g.^21–27^]. Early surveys on minke whales in the Southern Ocean included data on epibionts^28–37^, and several studies attempted to draw inferences on whale movements and population mixing based on infestation patterns^33,35,38^. These surveys were conducted before the Antarctic minke whale was recognized as a distinct species; so it is not always easy to tell apart which epibiotic associations involved *B. bonaerensis*, the dwarf form of *B. acutorostrata*, or both species. Nonetheless, evidence suggests that *B. bonaerensis* is more abundant in these waters, south of 60ºS^4^. Furthermore, the surveys that focused on epibionts as movement markers relied on small, opportunistic samples^33,38^, or made critical assumptions that require empirical confirmation. For instance, Bushuev^35^ found significant differences in the prevalence of the barnacle *Xenobalanus globicipitis* between whales captured in Antarctic International Whaling Commission Areas I, III, and IV^39^. Bushuev^35^ reasoned that, if whales had acquired barnacles in tropical and sub-tropical areas, infestation patterns would provide an indirect indication that whales from each sector had wintered in separate areas. However, this inference relies on two untested assumptions, namely, that barnacles cannot be recruited at high latitudes, and that their lifespan is at least as long as the duration of whales’ seasonal migration. Detailed data on host specificity, life history, and environmental tolerance limits are always necessary for the proper use of epibionts as population markers [see, e.g.^40^ for the case of parasites].

In the present study, we provide the first comprehensive quantitative description of the epibiotic fauna of Antarctic minke whales and put it in a comparative context with regard to other whales in the Southern Ocean and other regions. The potential influence of host-related factors (host body length, sex, and pod size) on settlement patterns is also examined. In addition, we investigate the extent to which recruitment of each epibiotic taxon occurs in the Southern Ocean (vs. in wintering grounds) based on rough estimates of the time elapsed since settlement [see^41^]. Overall, these data are used to assess the potential of each epibiotic taxon as a cetacean ecological marker. Special emphasis is put on *X*. *globicipitis* because of its apparent sensitivity to cold temperature ^35,42^ and high detectability under field conditions. In this case, latitudinal and thermal trends are also explored based on previous records of this species worldwide. Overall, this study is intended to serve as a basis to (i) understand the ecology of whale epibionts in the Southern Ocean and areas of putative recruitment and (ii) use epibionts to shed light on Antarctic minke whale long-distance movements.

## Methods

### Sample collection

**Figure 1:**
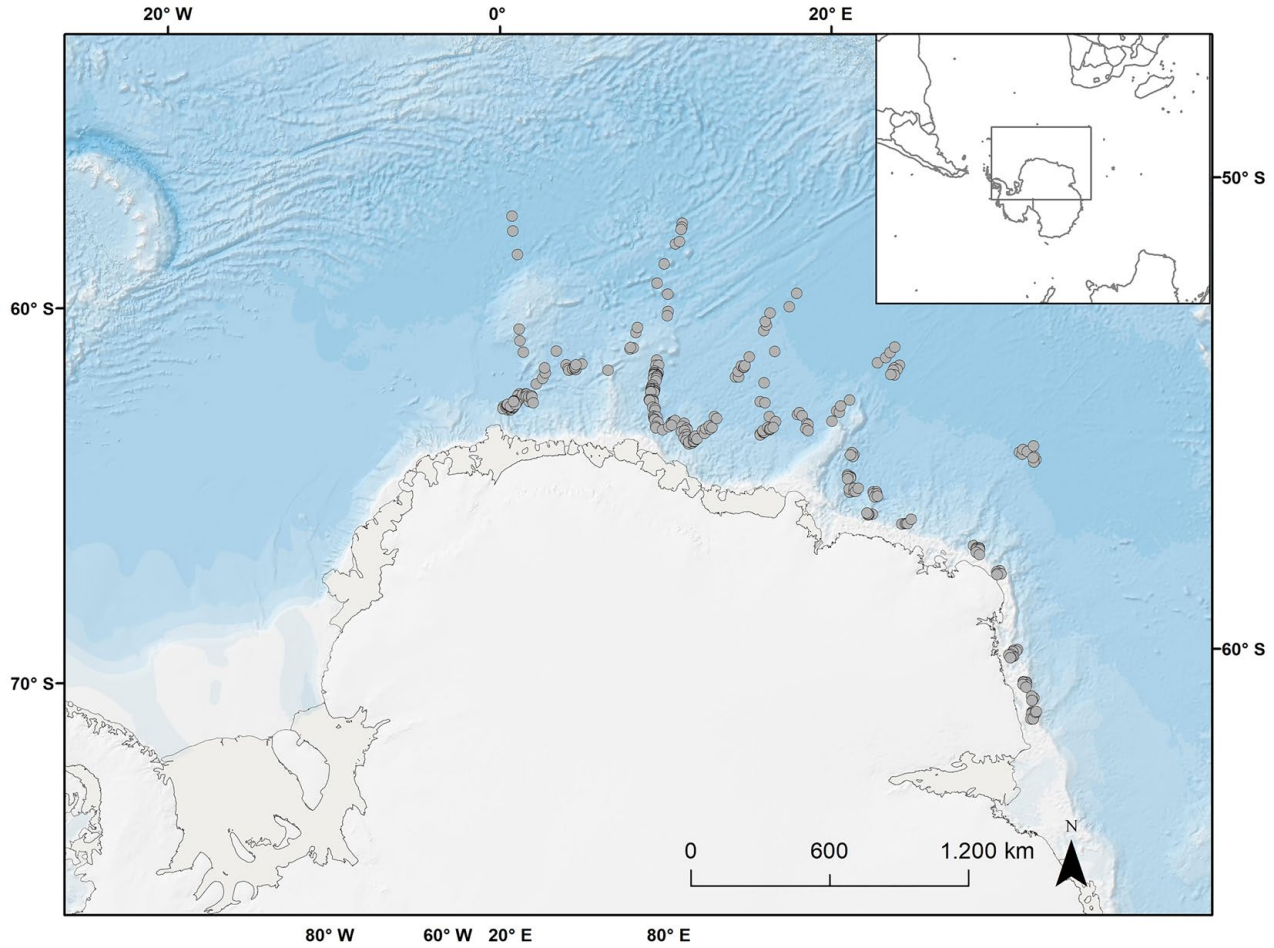
Study area. Gray dots indicate the location of each of the 333 Antarctic minke whales, *Balaenoptera bonaerensis*, captured in the Southern Ocean between December 2018 and February 2019. Figure generated with ArcGIS (version 10.8.1).

A total of 333 Antarctic minke whales (147 females and 186 males) were collected during the fourth and last survey of Japan’s New Scientific Whale Research Program in the Antarctic Ocean (NEWREP-A), a former research program. All experimental protocols were approved by the Government of Japan and all methods were carried out in accordance with the provisions of Article VIII of the International Convention for the Regulation of Whaling (ICRW). Sampling was carried out from December 18th to February 27th, 2018–19, in IWC Area III (0°–70°E), south of 60°S (Fig. 1), where water depth varies from 2000 to 5000 m and mean surface temperature (± SD) is 0.1 ± 0.9 °C during the austral summer (range: 1.9–2.4 °C). The number of whales collected in December, January, and February were 72, 163, and 98, respectively. The NEWREP-A program in 2018–19 was conducted under the permit “H30Suikan1735” issued by the Fishery Agency of Japan.

One or two whales from each sighted school were captured using a random sampling procedure^43^. Pod size was determined in all cases sensu IWC^44^. Most whales were observed to swim alone, but also in pairs or in groups of up to 12 individuals (mean ± SD = 1.7 ± 1.3). After capture, each whale was weighed and measured onboard to the nearest 0.01 MT and cm, respectively. The sample included adults and subadults with body weight ranging from 1.5 to 11.3 MT (mean ± SD = 6.0 ± 1.9) and length from 507 to 974 cm (800 ± 100).

**Figure 2:**
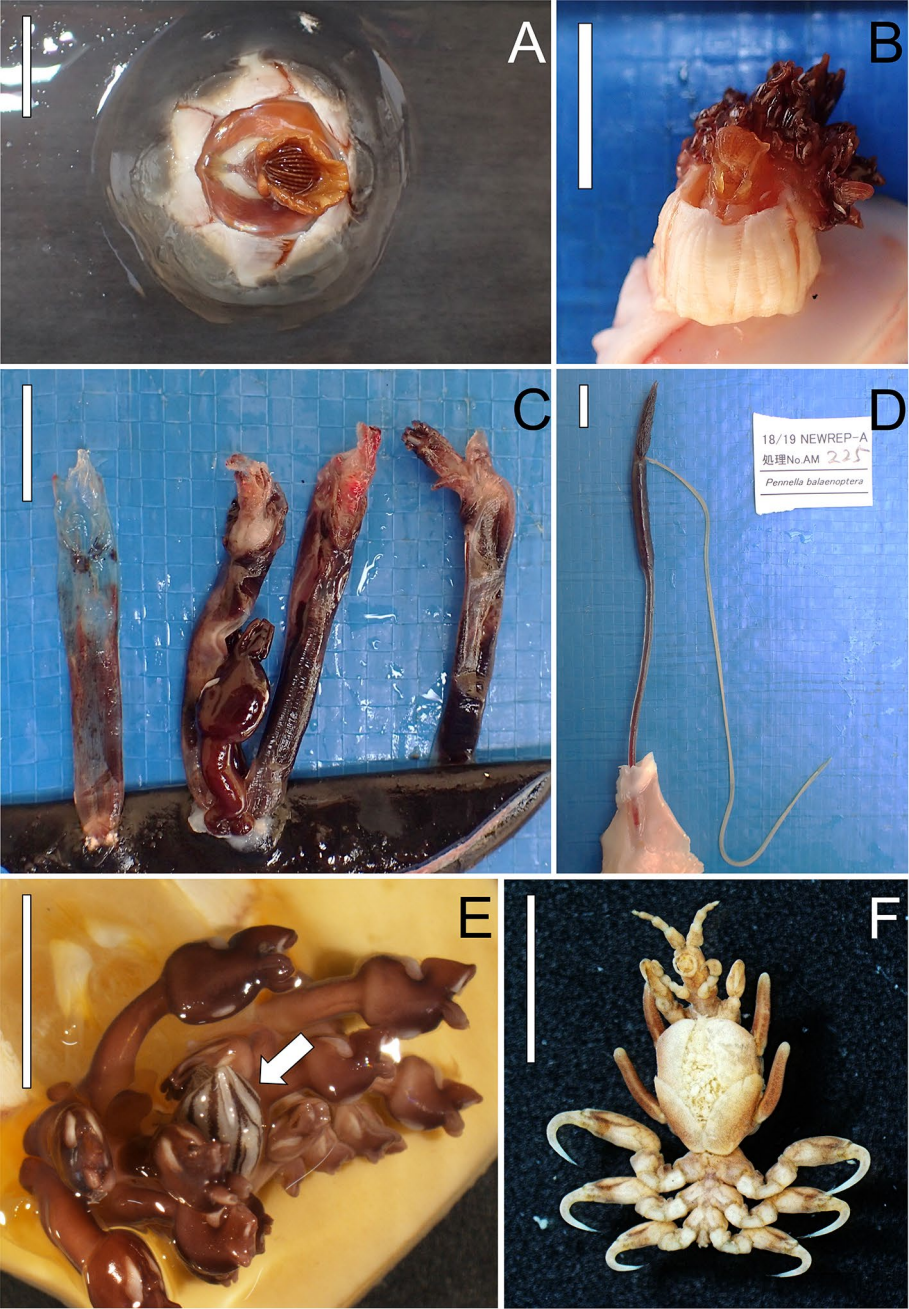
Epibiotic fauna of the Antarctic minke whale, *Balaenoptera bonaerensis*, captured in the Southern Ocean. (**A**) *Coronula reginae*, (**B**) *Coronula diadema* (with *Conchoderma auritum* attached to the shell), (**C**) *Xenobalanus globicipitis* showing a variable degree of tissue degradation (the leftmost specimen was scored as degree 2 and the three remaining ones as degree 1; see the text) and one individual of *C. auritum* on one shell, (**D**) female of *Pennella balaenoptera* with egg strings, (**E**) a single individual of *Conchoderma virgatum* (arrow) among individuals of *C. auritum* on the shell of *C. reginae*, (**F**) female of *Balaenocyamus balaenopterae* with offspring in the marsupium. Scalebar: 1 cm.

For each whale, the skin and pectoral flipper of either the right or left body side (depending on the particular placement of the animal onboard), as well as the tail flukes, dorsal fin, natural orifices (i.e. blowhole, anus, and genital slit), and baleen plates were examined for epibionts. The intensity (number of epibionts of a particular species on a single whale^45^) of each obligate epibiotic species (see “Results”) was approximated with an Intensity Estimate (IE) score: ‘1’ for 1–20 individuals, ‘2’ for 21–100, and ‘3’ for 101–500. Epibionts were also individually counted when IE = 1. The IE of the facultative barnacles *Conchoderma* spp. was not recorded because of the difficulties of counting minute individuals (Fig. 2) during the survey, and the limited utility of these barnacles as tags since they require obligate epibiotic barnacles for settlement [e.g.^46^; see the “Discussion”]. Voucher specimens of each epibiotic species were photographed, collected, preserved in 70% ethanol, and stored at the Institute of Cetacean Research (Japan).

### Species associations and predictors of epibiosis

For each epibiotic species, we calculated the prevalence (percent of whales harboring it) and set 95% Confidence Intervals (CI) using Sterne’s exact method^47^. We also calculated median abundance and range for each epibiotic species when the IE = 1. Pairwise associations between epibiotic species were tested with exact Chi-square tests for independent occurrence^45^.

Generalized Additive Models (GAMs), assuming a binomial distribution, were used to investigate the potential influence of sampling date and whale body length and sex, on the occurrence (i.e. presence/absence) of the 4 epibiotic species with a prevalence > 8%. Interactions were not included because models were exploratory. ‘Sex’ was included in the models as a fixed parametric factor, given that it has been proposed that minke whales have a sex-segregated distribution pattern in the Antarctic^48^. ‘Sampling date’ was considered as a proxy for the time spent by each whale in Antarctic waters, assuming that most whales had migrated to the Southern Ocean by December^11^. The date of first capture (December 18, 2018) was set as day 1. Due to the high number of absences (zeroes), we used ‘cloglog’ as the link function^49^. All explanatory variables were firstly tested for outliers and collinearity^49^. In the case of *Balaenocyamus balaenopterae*, which is the only species transmitted by whale-to-whale contact^50^, we also investigated the putative effect of pod size on its probability of occurrence. This predictor was highly collinear with body length; thus it was investigated alone in a separate model. Backward selection of the best model for each epibiont was made based on the lowest Akaike Information Criterion (AIC)^49^. Successful convergence was checked in all models to ensure that penalized cubic regression splines had sufficiently reduced the effects of concurvity between explanatory variables, if present^51^.

When whale length was found to be a significant predictor of occurrence, we also used one-way ANOVAs with polynomial contrasts to test for a significant trend in the length of whales at increasing IE. We also tested the influence of the sampling date on IE with a one-way ANOVA. For the only species with complete counts of individuals in all whales, i.e. *Coronula reginae*, we examined the relationship between exact epibiont intensity and whale length with a Spearman’s rank correlation test.

### Indicator potential

We investigated to what extent recruitment or reproduction of epibionts from Antarctic minke whales occurred in the Southern Ocean based on the available criteria found in the literature for each species (Table 1). To this end, we examined pictures of a subsample of epibionts obtained in situ from the whales, and of voucher specimens collected during previous whaling seasons (1990–2018) and stored at the Institute of Cetacean Research (ICR), Tokyo. The imaging software *Fiji*^52^ was used to obtain morphometric measurements as follows: total length without antennae of *B. balaenopterae* (n = 4 specimens), capitulum length of *Conchoderma auritum* (n = 334) and *C. virgatum* (n = 1), abdomen length and distal width of *P. balaenoptera* (n = 2), and maximum shell diameter for both *Coronula* spp. (n = 55) and *Xenobalanus globicipitis* (n = 89). Only the exposed part of *P. balaenoptera* and *Coronula* spp. was measured since they partly embed in hosts’ skin. Gross estimations of time since settlement were also made whenever possible based on published data on growth rates of relevant structures (see Table 1).

**Table 1:** Empirical criteria used to estimate recruitment or reproduction of the epibiotic fauna found on Antarctic minke whales, *Balaenoptera bonaerensis*, in the Southern Ocean.

Species	Criterion	Inference	Reference
**Amphipoda**			
*Balaenocyamus balaenopterae*	Eggs or young in marsupium	Active reproduction	^110,111^
**Cirripedia**			
*Xenobalanus globicipitis*	Shell shape round	Newly recruited individual	^57,148^
	Maximum diameter of shell > 3.67 mm	Adult	
*Coronula* spp.	Diameter of non-embedded shell: 5–10 mm	Young individual	^75^
	Diameter of non-embedded shell: 25–50 mm	Adult	
*Conchoderma* spp.	Capitulum length < 2 mm	Newly recruited individual	^112–114,118,119,149–151^
	Capitulum length > 7 mm	Most *C. auritum* carrying embryos	
	Capitulum length > 8.8 mm	Adult of *C. virgatum*	
	Growth rate: 0.1–1.5 mm/day	Time since settlement	
**Copepoda**			
*Pennella balaenoptera*	Length of non-embedded abdomen > 93.3 mm	Adult	^57^
	Presence of egg strings	Active reproduction	^152^

In the case of *X. globicipitis*, we carried out additional analyses because all individuals were found dead at the time of collection, showing a variable degree of tissue degradation (Fig. 2C). It is thus likely that this barnacle, which attaches exclusively to cetaceans [e.g.^53^], cannot withstand the environmental conditions of Antarctic waters. This opened three additional avenues to explore the potential of *X. globicipitis* as a migration marker. Firstly, we investigated the geographical limits and thermal tolerance of this species. To this end, we adopted the systematic approach described in Ten et al.^27^ to obtain all the available records of the presence/absence of *X. globicipitis* worldwide based on publications that reported the examination of the external surface of cetaceans. For each selected survey, we also compiled the sampling coordinates (to the nearest 0.1°) and sampling period. Then, we used a binomial GAM with a ‘logit’ link function to model the relationship between the presence/absence of *X. globicipitis* and the minimum Sea Surface Temperature (SST, °C) prevailing in the sampling localities and periods. SST values were extracted with Python 3.10.2 from the ESA SST CCI and C3S global Sea Surface Temperature Reprocessed product, held by the E.U. Copernicus Marine Environment Monitoring Service (CMEMS) (https://doi.org/10.48670/moi-00169). To avoid the unduly influence of extreme SST values, we used the 10th percentile of average annual SST (SST_p10_), averaged for the sampling period and coordinates (including all points within a 0.5° radius) of each survey. Since the first full year of CMEMS dataset is 1982, the SST_p10_ values for earlier surveys were imputed as average values from the period 1982–1986.

We excluded the records of fourteen cetacean species because they perform long latitudinal migrations between polar and tropical regions, thus the actual localities of epibiont recruitment are uncertain and could lead to biased results. Nonetheless, we explored whether these data (i.e. migrating cetaceans) followed a similar trend to those included in the GAM with a chi-square test (presence/absence between 2 categories: those sampled within 50ºN-40ºS or those sampled outside this range; see “Results”). It is important to note that some species do not or do rarely harbor *X. globicipitis*, and this could be due to factors other than geographical range or migrations (e.g. cetacean hydrodynamics; Ten et al. unpubl.).

Secondly, we carried out a gross estimation of the number of recruitment events of *X. globicipitis* to each Antarctic minke whale based on the similarity of the relative age of the barnacles it harbored. The maximum shell diameter (MSD) is considered an acceptable proxy for relative age in *X. globicipitis*^54–56^. To “gauge” estimations, we visually compared box-and-whisker plots of MSD, for a given barnacle abundance per host, between Antarctic minke whales and a sample of infested striped dolphins, *Stenella coeruleoalba*, from the western Mediterranean, for which recruitment of *X*. *globicipitis* seems not spatially or seasonally restricted^56,57^. We obtained MSD measurements from 89 individuals of *X*. *globicipitis* from 15 whales (mean no. of barnacles ± SD per whale: 6.3 ± 4.2 ind./host, range 1–15) and 270 individuals from 46 striped dolphins (5.7 ± 4.2 ind./host, range 1–15) stranded all year round.

Finally, we tested whether the degree of tissue degradation of dead individuals of *X. globicipitis* on Antarctic minke whales could be used as an indicator of latitude and/or time spent by whales in the Southern Ocean. We assigned a ‘degradation score’ to each of 677 photographed specimens from 36 whales (mean no. ± SD = 18.8 ± 24.1 individuals/whale) using the following criteria (Fig. 2C): ‘1’, whole specimen; ‘2’, partial remains of the specimen; ‘3’, only the shell; and ‘4’, only the scar left by the shell. We then performed a Spearman’s rank correlation test whether the average degradation score per whale increased with sampling date. For future indicator purposes, we also considered just two categories that could be verified in the field, namely, ‘1’, whole or degraded prosoma observed in at least one barnacle on the whale; ‘2’, only shell(s) and/or some empty cuticle observed. Mean sampling date and 95% bootstrap confidence interval with 10,000 replicates were calculated for each of these two groups.

All analyses were performed with R (version 4.1.2; R Core Team, 2021). GAMs were conducted with the ‘mgcv’ R package and models were fitted based on the generalized cross-validation criterion (GCV), which automatically selects the effective degrees of freedom and controls for oversmoothing^58^. Penalized cubic regression splines were set for smoothing and diagnostic plots of residuals were checked to examine model suitability^59^. For all tests, statistical significance was set at p < 0.05. 

## Results

Seven epibiotic species were found, including the ectoparasitic amphipod *Balaenocyamus balaenopterae*, the mesoparasitic copepod *Pennella balaenoptera*, three obligate commensal barnacles of the family Coronulidae, i.e. *Xenobalanus globicipitis*, *Coronula reginae*, and *C. diadema*; and two facultative commensal barnacles, i.e. *Conchoderma auritum* and *C. virgatum* (Fig. 2). Out of the 333 whales analyzed, 125 (37.5%) harbored at least one epibiont, with a maximum of 4 species. The number of whales with 4, 3, 2 and 1 epibiotic species were 2, 12, 19 and 92, respectively. The sex ratio of the infected hosts was similar for all epibionts with prevalence > 1%: *B. balaenopterae* (on 37 females/37 males), *X. globicipitis* (14/23), *C. reginae* (13/16), and *C. auritum* (13/17). The rest of species settled on whales of the same sex: *C. diadema* (on 3 females), *P. balaenoptera* (on 2 males), and *C. virgatum* (on 1 male).

Prevalence was < 25% for *B. balaenopterae* and *X. globicipitis*, and < 10% for the remaining species (Table 2). Infected whales harbored few individuals (IE = 1) of each species, except *B. balaenopterae* and *X. globicipitis*, for which IEs of 2 or 3 were found in 52.7% and 64.9% of infected whales, respectively (Table 2). All epibiotic species occurred on defined microhabitats except *C. reginae*, which was found covering the whole body (Table 2). *Conchoderma auritum* was found attached to the shell of *C. reginae* (on N = 19 whales), *C. diadema* (N = 2), and *X. globicipitis* (N = 7), and to the baleen plates of a single whale devoid of coronulid barnacles; *C. virgatum* was found settled on *X. globicipitis* on a single whale. All barnacles exhibited positive associations that were statistically significant except for that between *C. diadema* and *X. globicipitis* (Table 3). In contrast, the associations between *B. balaenopterae* and all barnacle species were negative, with those involving *X. globicipitis* and *C. auritum* being significant (Table 3).

**Table 2:** Infestation parameters and microhabitat of the epibiotic fauna found on Antarctic minke whales, *Balaenoptera bonaerensis*, in the Southern Ocean.

Species	Microhabitat	Prevalence (95% CI)	IE (n)	Median [range]
**Amphipoda**				
*Balaenocyamus balaenopterae*	Ventral grooves and genital slit	22.2 (18.0–27.0)	1 (35)	6 [1–19]
			2 (26)	
			3 (13)	
**Cirripedia**				
*Coronula reginae*	Ubiquitous	8.7 (5.8–12.0)	1 (29)	2 [1–13]
*Coronula diadema*	Ventral side	0.9 (0.2–2.6)	1 (3)	1
*Conchoderma auritum*	On coronulid barnacles, baleen plates (1 case)	9.0 (6.3–12.6)	–	
*Conchoderma virgatum*	On *X. globicipitis*	0.3 (0–1.7)	–	
*Xenobalanus globicipitis*	Flukes and pectoral fins	11.1 (8.1–15.0)	1 (13)	14 [1–20]
			2 (22)	
			3 (2)	
**Copepoda**				
*Pennella balaenoptera*	Flanks	0.6 (0.1–2.2)	1 (2)	1

‘IE’ is an Intensity Estimate where 1 stands for 1–20 specimens, 2 for 21–100, and 3 for 101–500; ‘n’ is the number of whales included in each category. The median number and range are provided whenever IE = 1. In the case of *Conchoderma* spp., IEs were not obtained.

**Table 3:** Pairwise chi-square tests for co-occurrence of the epibiotic fauna found on Antarctic minke whales, *Balaenoptera bonaerensis*, in the Southern Ocean.

	*Bb*	*Cr*	*Cd*	*Xg*	*Ca*
*Balaenocyamus balaenopterae*	–	2.34 ( −)	0.93 ( −)	4.79 ( −)	4.62 ( −)
*Coronula reginae*	0.157	–	55.3 ( +)	13.69 ( +)	113.95 ( +)
*Coronula diadema*	0.591	** < 0.001**	–	0.41 ( +)	31.21 ( +)
*Xenobalanus globicipitis*	**0.029**	**0.001**	0.999	–	103.03 ( +)
*Conchoderma auritum*	**0.036**	** < 0.001**	** < 0.001**	** < 0.001**	–

The values of the χ2 statistic, and associated exact probability (with significant values marked in bold), are shown in the upper and lower triangular matrices, respectively. Column headings correspond to epibiotic species' names; (+) and (−) stand for positive and negative association, respectively.

‘Whale body length’ was included as a positive predictor in the final GAM models of occurrence of the four epibiotic species (Supplementary Fig. 1), but the deviance explained was low (< 8%) in all cases (Table 4). The effect was statistically significant for *B. balaenopterae* (p < 0.001) and *C. reginae* (p = 0.029), and close to significance for *X. globicipitis* (p = 0.052) and *C. auritum* (p = 0.072) (Table 4). Considering infected whales only, we failed to find a significant positive trend of host body length or sampling date at increasing IE for *B. balaenopterae* (one-way ANOVA, all polynomial contrasts with p > 0.175), or a positive correlation between host length and the number of *C. reginae* (r_s_ = − 0.05, n = 28, p = 0.803). ‘Sampling date’ was also included as a negative predictor in the final models for the three barnacle species, but the effect was found to be close to significance only for *X. globicipitis* (Table 4, Supplementary Fig. 1). Finally, we did not detect a significant influence of ‘host sex’, or ‘pod size’ in the case of *B. balaenopterae*, and these predictors were also excluded from the models with the lowest AIC.

**Table 4:** Stepwise selection of binomial models based on the lowest value of Akaike Information Criterion (AIC).

Species	Model structure	AIC	Adj-R^2^	Dev (%)
*Balaenocyamus balaenopterae*	Presence ~ s(WBL) + s(SD) + S	338.76	0.0478	7.11
	Presence ~ s(WBL) + S	337.31	0.0318	4.00
	Presence ~ s(WBL)	336.23	0.0327	3.73
	Presence ~ s(Pod size)	347.62	0.0103	3.15
*Conchoderma auritum*	Presence ~ s(WBL) + s(SD) + S	202.67	0.0140	3.45
	Presence ~ s(WBL) + s(SD)	200.76	0.0165	3.41
	Presence ~ s(WBL)	201.35	0.0086	2.12
*Coronula reginae*	Presence ~ s(WBL) + s(SD) + S	186.73	0.0284	7.32
	Presence ~ s(WBL) + s(SD)	184.93	0.0297	7.56
	Presence ~ s(WBL)	185.90	0.0208	5.82
*Xenobalanus globicipitis*	Presence ~ s(WBL) + s(SD) + S	230.21	0.0224	5.61
	Presence ~ s(WBL) + s(SD)	228.34	0.0260	5.57
	Presence ~ s(WBL)	233.73	0.0039	1.12

The presence (or absence) of the four more prevalent epibiotic species on Antarctic minke whales, *Balaenoptera bonaerensis*, is investigated with three predictors: *WBL* whale length, *SD* sampling date, and *S* sex of the whale. The effect of pod size was tested only for *Balaenocyamus balaenopterae* (see main text). Other values for model evaluation include the R^2^-adjusted coefficient and the percentage of deviance explained.

Inferences about recruitment and/or active reproduction of all epibiotic taxa are summarized in Table 5 (all measurements in Supplementary Table 1). We detected indications of active recruitment on whales for *C. auritum* (over 35% of the whales harbored barnacles with a capitulum < 2 mm), and of active reproduction for *B. balaenopterae* (2 females in the sample of voucher specimens harbored offspring in the marsupium) and *P. balaenoptera* (the two females found in the 2018–19 sample carried egg strings) (Fig. 2D,F). The two specimens of *C. virgatum* could be classified as adult (capitulum length > 15 mm) or late juvenile (> 7 mm), respectively. The sample of *Coronula* spp. included putative adult and juvenile barnacles, but only one individual (of *C. reginae*) exhibited a minute size (diameter of shell: 2.9 mm) suggestive of recent recruitment. Likewise, we detected no newly recruited individuals in the sample of 91 *X. globicipitis* whose shell could be measured. Moreover, no *X. globicipitis* from the overall 2018–19 sample (n = 677) was alive when detected, showing variable degrees of tissue degradation (Fig. 2C).

**Table 5:** Inferences of recruitment, age classes and reproductive status of the epibiotic fauna found on Antarctic minke whales, *Balaenoptera bonaerensis*, in the Southern Ocean.

Species	Criteria	Measured subsample		Measured subsample (per whale)		Voucher specimens	
		n	Inference	N	Inference	n	Inference
*Balaenocyamus balaenopterae*	Brood in pouch	–	–	–	–	66	2 FY
*Coronula reginae*	MSD	54	J: 18.5	16	WJ: 37.5	18	J: 0
			A: 16.7		WA: 37.5		A: 72.2
*Coronula diadema*	MSD	1	J: 100	1	WJ: 100	–	–
			A: 0		WA: 0		
*Conchoderma auritum*	CL	334	NR: 8.4	18	WNR: 37.5	36	NR: 5.6
			A: 21.9		WA: 66.7		A: 5.6
*Conchoderma virgatum*	CL	1	NR: 0	1	WNR: 0	1	NR: 0
			A: 100		WA: 100		A: 0
*Xenobalanus globicipitis*	MSD	89	NR: 0	15	NWR: 0	2	NR: 0
			A: 39.3		A: 53.3		A: 0
*Pennella balaenoptera*	Body size and presence of egg strings	2	A: 100	2	A: 100	1	A: 0
			FE: 100		WFE: 100		FE: 0

Inferences were drawn from a subsample of *n* measured epibionts, from the measured epibionts of each whale (*N* whales), and from additional voucher specimens deposited at the Institute for Whale Research of Japan. (see also Table 1): *A* Percent adults, *CL* Capitulum Length, *FE* Percent females with egg strings, *FY* No. females with young in marsupium, *J* Percent juveniles, *MSD* Maximum Shell Diameter, *NR* Percent newly recruited individuals, *WA* Percent whales harboring adults, *WFE* Percent whales harboring females with egg strings, *WJ* Percent whales harboring juveniles, *WNR* Percent whales harboring newly recruited individuals. See text for measurement specifications.

**Figure 3:**
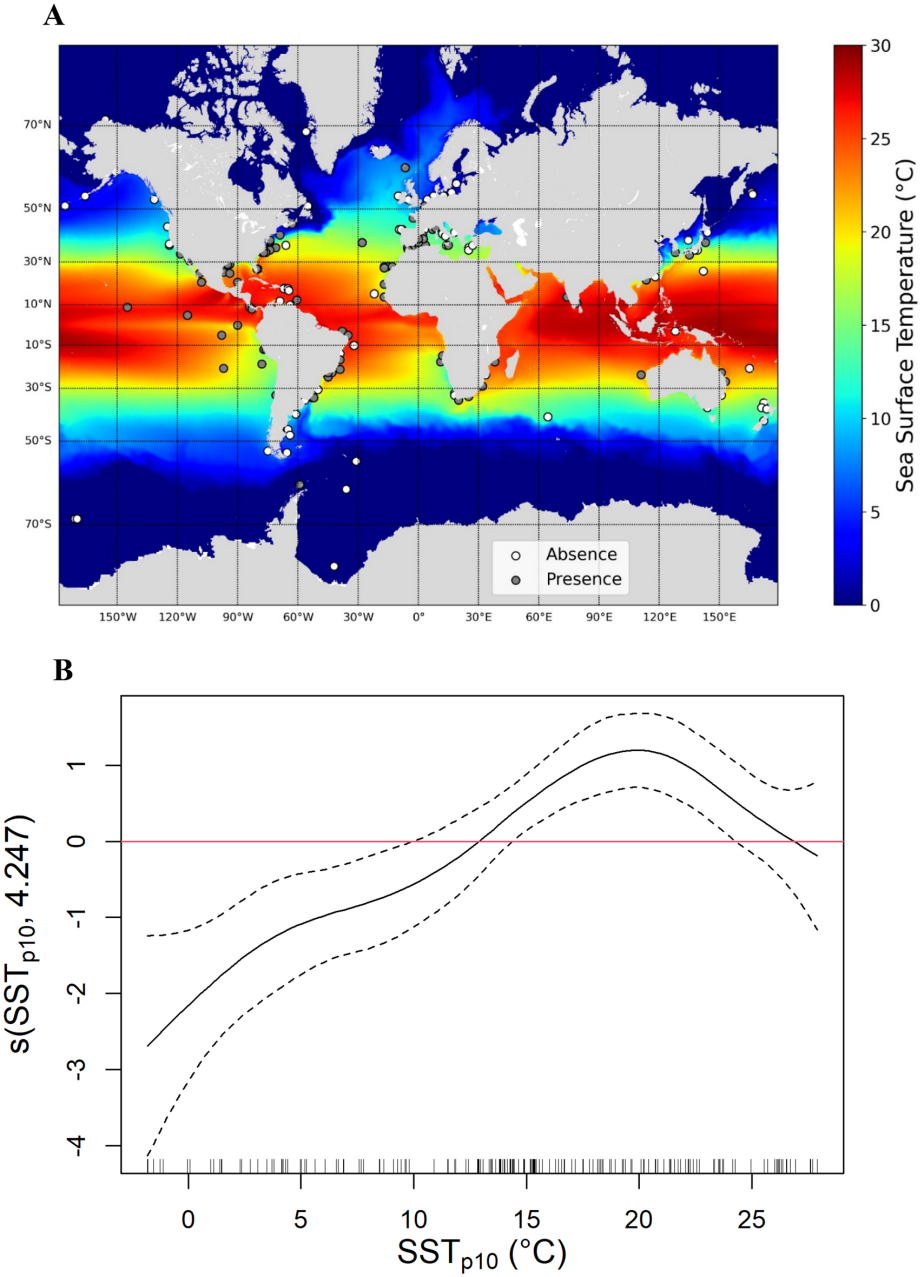
Latitudinal and temperature trends on the cetacean barnacle *Xenobalanus globicipitis*. (**A**) Data points obtained from a review of publications examining the external surface of cetaceans. Gray dots indicate presence of *X. globicipitis*, whereas white dots indicate absence. The color gradient indicates minimum sea surface temperature (SST_q10_, see “Materials and Methods”). This figure was generated with Python (version 3.10.2). (**B**) Estimated smoother for the binomial GAM used to model the probability of *X. globicipitis* presence (data obtained from a literature review) as a smooth function of SST_q10_. The solid line is the estimated smoother and the dotted lines are 95% point-wise confidence bands. Places where the confidence bands are wide and enclose the horizontal red line indicate values where the overall pattern is not significant^49^. The y-axis shows the contribution of the smoother, which is centered around zero, to the fitted values.

GAMs included 276 data points of the presence of *X. globicipitis* on 48 cetacean species. Data were compiled from 192 pieces of literature (including peer-reviewed articles and gray literature; Supplementary Table 2). All records of *X. globicipitis* had latitudinal limits around 50ºN-40ºS, corresponding to isotherms of *c.* 15ºC, except for long-finned pilot whales, *Globicephala melas*, around Faroe Islands (NE Atlantic) and South Shetland Islands (SW Atlantic) (Fig. 3A; Supplementary Table 2). Note, however, that the first exploit different areas in the Northeast Atlantic^60^ and the second seem to also move along a relatively broad latitudinal range across the Southern Hemisphere^61^. GAMs indicated that the probability of finding *X. globicipitis* on cetaceans was higher at warmer temperatures (p = 2e-^16^, R^2^(adj) = 0.18, deviance explained = 15.2%, N = 276). A marked drop was observed when minimum temperatures were lower than ~ 12ºC (Fig. 3B). Places where the confidence bands are wide and enclose the horizontal red line indicate values where the overall pattern is not significant^49^. The trend in cetacean species performing long-distance migrations (Supplementary Table 3), excluded from the previous analysis to avoid potential biases, was similar: it was less likely to spot *X. globicipitis* outside 50ºN-40ºS (p = 8.8e-^5^, χ^2^ = 15.4, N = 211).

**Figure 4:**
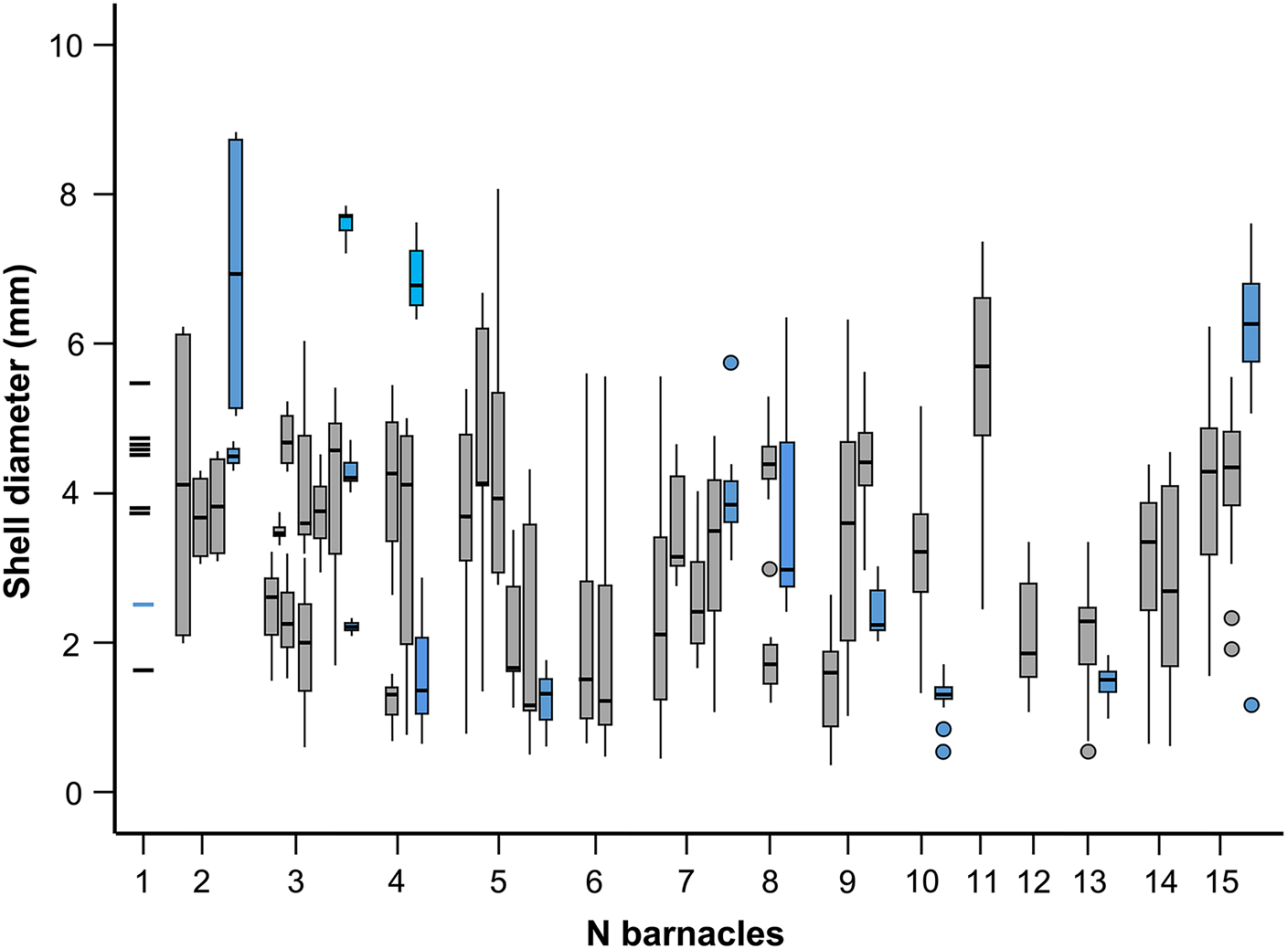
Box-and-whisker plots showing the maximum diameter of the shell of populations of the barnacle *Xenobalanus globicipitis* on 15 Antartic minke whales, *Balaenoptera bonaerensis* (blue boxes) from the Southern Ocean and 44 striped dolphins, *Stenella coeruleoalba* (gray boxes), from the western Mediterranean.

Specimens of Antarctic minke whales had larger MSD (mean ± SD = 3.46 ± 2.26 mm, range = 0.53–8.70; N = 88) than those of Mediterranean striped dolphins (2.75 ± 1.53 mm, 0.47–9.02; N = 455). Also, size variation on each whale was significantly lower in Antarctic minke whales (t(55) = -3.283, p = 0.002; Fig. 4). Lastly, the degree of degradation of *X. globicipitis* was positively correlated to sampling time (r_s_ = 0.359, p = 0.032) and whales presenting only shells of *X. globicipitis* (simplified score ‘2’) were captured, on average, later in the season (i.e. mean day = 31.0 [95% CI = 25.6–36.4]); although differences were subtle (i.e. 27.0 [21.6–32.4] for score ‘1’).

## Discussion

Except *Coronula diadema*, the epibiotic species found in the present survey had already been reported in the Southern Ocean, on minke^33,35–37^ and other baleen whales [e.g.^42,62,63^]. Moreover, these epibionts are apparently cosmopolitan^27^ and, except *Coronula* spp., have also been detected on common minke whales in the Northern Hemisphere^24,64,65^. Note, however, that identification of these species has hitherto been based largely on morphological evidence, and cryptic speciation is fairly common among marine invertebrates^66–68^, including epibionts [e.g.^69,70^]. Future molecular analyses could assist in exploring hidden diversity of these putatively cosmopolitan epibionts.

The epibiotic species found seem also generalist in their use of cetaceans as basibionts, although there are no detailed studies addressing the possibility of cryptic specificity [e.g.^71^]. The available evidence suggests that *B*. *balaenopterae* and the two *Coronula* spp. exhibit the most restricted host range. The former is an obligate skin-feeding ectoparasite associated to *Balaenoptera* spp. worldwide^27,72,73^. In most surveys, its prevalence is surprisingly low (< 10%)^27^ and higher values have only been reported in Antarctic minke whales: 20% in South Africa^33^, and 22% and 36% in the Southern Ocean [^36^, this study]. Reasons behind these figures are difficult to decipher, but the role of hosts’ population density should be considered since whale lice are transmitted by contact ^74^. In contrast, *Coronula* spp. are commensal barnacles with a free-living cyprid stage to colonize baleen whales^75–77^ and, rarely, large odontocetes [e.g.^78–81^]. Quantitative evidence strongly suggests that both *C. diadema* and *C. reginae* depend on humpback whales, *Megaptera novaeangliae*, as basibionts^27,82^ with prevalence being generally *ca.* 100% regardless of locality^62,75,79,83,84^. Apparently, the presence of these barnacles in other whales with low prevalence, including *B. bonaerensis* in this study (< 10%), would suggest geographical overlap with their main hosts, at least where larval release and recruitment occur^85^.

Four additional epibionts appear to be more generalist dwellers. The commensal barnacle *Xenobalanus globicipitis* is known to settle on over 41 cetacean species^27^, with no clear evidence of preferential attachment to particular host species. Nevertheless, this species rarifies in cold regions (see below) and, indeed, records in the Southern Ocean are scanty regardless of cetacean species. A few records exist from the beginning of the last century^28,42,86,87^, but the species was seemingly not found in the intense commercial whaling campaigns carried out in the 1950s^62,83,88–92^, being later reported with low to moderate prevalence [^35,93^, this study]. Future studies should clarify whether this striking pattern is a sampling artifact (i.e. we also considered individuals with the shell only). The mesoparasite *Pennella balaenoptera* is believed to use cephalopods as first hosts, in which it develops into an adult stage and, after mating, the inseminated female seeks cetaceans to attach and produce eggs^94^. This species is widespread in both mysticetes and odontocetes worldwide^27^, but the host spectrum of inseminated females could actually be wider since recent molecular evidence suggests that this species could be conspecific with *P. filosa*, which infects large oceanic teleosts^94^. The low prevalence found in Antarctic minke whales from this study agrees with previous figures (0–11.4%) obtained from other whales in the Southern Ocean^27^. Finally, the cosmopolitan barnacles *Conchoderma auritum* and *C. virgatum* are facultative epibionts on hard substrata which are able to dwell, not only on a number of marine animals, but also on flotsam and ship hulls^95–97^. In cetaceans, both species occasionally settle on teeth or baleen plates, but more regularly on other epibionts, i.e. *P. balaenoptera* and, especially, *Coronula* spp.^63,87,98–100^, as we found in Antarctic minke whales. However, the hyperepibiotic association of both *Conchoderma* spp. with *X. globicipitis* is reported here for the first time [see ^27^].

Our results suggest that whale’s body length was a poor but significant positive predictor of several of these epibionts. In the case of *B. balaenopterae*, it is possible that larger (= older) whales have had more opportunities for physical contact with infected counterparts. This relationship, however, was not previously observed by Bushuev^35^. Besides, we did not find evidence that pod size (a surrogate of more potential contacts) influenced the likelihood of finding *B. balaenopterae*. Moreover, one could expect that larger (= older) whales also harbor higher numbers of whale lice, but we failed to find such a trend [see also^24^]. Overall, these results suggest that the occurrence and number of whale lice on individual whales depend on a number of unknown factors affecting the number of physical contacts with other whales (via social interactions) and/or the growth rate of whale lice populations. With regard to the barnacle species, we also found very modest, positive relationships between their occurrence and whale size; and the patterns were statistically significant (*C. reginae*), or close to significant (*X. globicipitis, C. auritum*). It is unlikely that, in this case, larger (= older) whales have more chances to accumulate recruits over time because barnacles use a free-living (cyprid) larva to colonize whales and the adult stage has a relatively short lifespan, i.e. about 6 months for *X. globicipitis*^26,101,102^ and one year for *Coronula* spp.^76,103^. Perhaps larger whales simply offer more surface for initial cyprid contact, a point that merits further attention in future studies.

Our data shows contrasting evidence on epibiont attachment to Antarctic minke whales in the study area. In the case of the two parasites, we found convincing evidence of active reproduction, but not of recruitment. The two specimens of *P*. *balaenoptera* were large ovigerous females^104,105^ likely acquired by whales at lower latitudes; in fact, *P*. *balaenoptera* seems to be more prevalent in tropical or temperate waters^27^. Recruitment in Antarctic waters could only be proven if newly attached individuals had been found, but none of the few surveys reporting the size of *P. balaenoptera* in polar waters have hitherto supported this possibility^28,65,86,106–108^. In this respect, the epidemiology of *Pennella* sp. in the Pacific saury, *Cololabis saira*, is instructive: this oceanic fish overwinters in subtropical areas and migrates to subarctic waters in the summer^109^. Interestingly, fish infected with *Pennella* sp. were detected throughout the distribution range, but newly recruited parasites were detected only in the southern part of the distribution^109^, suggesting a positive link with more benign conditions and/or occurrence of intermediate hosts. Whether this also occurs in *P. balaenoptera* deserves a closer look.

Likewise, 2 out of 66 examined individuals (3%) of *B. balaenopterae* were females with young in the pouch. Although the sample size is modest, this percentage is not particularly small when compared with detailed data from other whale lice^110^. However, whether new generations of *B. balaenopterae* are produced and released from the pouch (i.e. recruited) during the stay of minke whales in Antarctica remains unknown. We did not find evidence that the likelihood of occurrence nor intensity of *B. balaenopterae* changed throughout the spring–summer, thus opposing the hypothesis of extensive recruitment. In other whale lice infecting migrating whales, i.e. *Cyamus scammoni* and *C. kessleri* from grey whales, *Eschrichtius robustus*, the schedule of development and reproduction appears to be protracted and variable but, in agreement with our observations, recruitment of young lice is rare in the summering grounds^111^.

In the case of the facultative epibiotic barnacles, it is unclear whether recruitment of *C. virgatum* may occur because all individuals were putative adults, but our data indicate that at least 8% of *C*. *auritum* had recently attached to whales since specimens with capitulum length ~ 2 mm were found throughout the study period. The growth rate of the capitulum of newly settled *C*. *auritum* is very fast during the early stages of development, ~ 0.5–1 mm/day^112–114^. It is possible that the low temperatures in the study area delayed growth [see^115^]; in fact, the mean and maximum values of capitulum size were small compared to those from individuals of *C*. *auritum* collected in temperate waters^116^. However, it is extremely unlikely that whales had maintained alive minute individuals of *C*. *auritum* during the long southbound migrations that take at least two months^11,117^. In any event, it seems clear that both larval dispersion^46^ and settlement of *C*. *auritum* are frequent in temperate and tropical waters^97,112,114,118,119^, thus recruitment of this species may also have occurred on migrating Antarctic minke whales.

Our data strongly suggest that none of the coronulid barnacles specific to cetaceans recruits regularly in the study area. The exposed shell of *Coronula* spp. in our sample ranged from c. 3 to 28 mm. These values seem smaller than those from specimens collected in their main hosts, i.e. humpback whales, at lower latitudes (total shell diameter: 40–85 mm)^46,82,120^, thus raising the question of whether Antarctic minke whales could be suboptimal hosts. In any event, sizes are large enough to rule out recent attachment [see ^75^]. At least in the case of *C. diadema*, a one-year life cycle has been proposed^76,103^: barnacles would settle on whales in their wintering grounds, grow during whales’ spring migrations and in the summer grounds at high latitudes, and then reproduce and die in the autumn migration and in the wintering grounds^63,75,85,86,117–124^. Accordingly, the occurrence of putative young and adult individuals of *Coronula* spp. in our sample would be best interpreted as the results of previous settlement in late winter or early spring.

Most strikingly, all individuals of *X. globicipitis* in our sample were dead and exhibited variable degrees of tissue degradation. This suggests that this species cannot withstand the low temperatures prevailing in the polar realm. In fact, its likelihood of occurrence decreased (albeit not significantly) and the degree of degradation increased along the season, indicating that whales were losing the barnacles previously recruited at lower latitudes. Furthermore, the number of inferred recruitment events based on the size of barnacles was clearly lower in minke whales than in a cetacean species for which recruitment of *X. globicipitis* is probably not seasonally restricted, i.e. Mediterranean striped dolphins^57^. Most importantly, GAMs revealed a sharp drop in the odds of finding *X. globicipitis* when minimum temperatures were lower than ~ 12 °C. This result provides, for the first time, quantitative support to the long-standing claim that *X. globicipitis* recruits in temperate and tropical regions [e.g.^25,35,125^]. Therefore, its populations are probably maintained by cetaceans permanently living in temperate and tropical waters.

## Conclusions

Several key inferences can be drawn from the previous analyses. Firstly, *B. balaenopterae*, transmitted by contact, will be useful for phylogeographic studies, potentially shedding light on intra- and interspecific interactions between whale individuals or populations [see e.g.^23,126,127^]. Moreover, the potential relationship of *B. balaenopterae* prevalence with the population density of balaenopterid whales in the Southern Ocean opens interesting venues to analyze historical whaling data. For instance, perhaps it is not coincidental that the lowest prevalences of *B. balaenopterae* have been recorded in the most heavily exploited species [see e.g.^62,83,88–92^].

Secondly, our data suggest that *C. auritum* can be apparently recruited in the Southern Ocean, but its colonization strongly depends on the co-occurrence of other epibionts, especially coronulid barnacles, on which to settle; this explains why we found a strong and significant association with them. This circumstance greatly limits the value of *C. auritum* as an independent indicator.

Thirdly, the detection of any of the 3 coronulid barnacles and, possibly, the copepod *P. balaenoptera* in Antarctic minke whales could inform on some aspects of their ecology. On the one hand, significant, consistent differences in the prevalence of this subset of epibiont species between Areas of the Southern Ocean will inform about the existence of whale stocks that (i) winter in different localities and (ii) do not mix extensively in the summer. On the other hand, these epibionts could allow to obtain gross estimations of the minimum portion of migrating Antarctic minke whales to lower latitudes outside the Southern Ocean.

Finally, coronulid barnacles, and especially *X. globicipitis*, could shed additional light on wintering areas and the timing of migrations. Interestingly, we observed that these barnacles tend to co-occur in the same whales, which suggests that there are ‘hot spots’ for recruitment of these species at low latitudes, as Dreyer et al.^128^ recently suggested [see also^25,129^]. In the case of *X. globicipitis*, our results also reveal an area of higher probability of cetacean colonization within 50°N-40°S, especially in regions where minimum SST is 15-20 °C. Indeed, all cetacean species harboring this barnacle exploit, at least partially, waters within the 30°N-30°S belt [see^27^]. In this context, Kasamatsu et al.^11^ inferred that breeding areas of Antarctic minke whales might be located between 5° and 30°S, and neonates and calves have been spotted within 2-34°S ^18,19,33,130–132^. Accordingly, the detection of *X. globicipitis* could be a strong indication of minke whale stays in this latitude range, as some authors have previously suggested^24,25,35,125,129^. In any event, the value of both *Coronula* spp. and *X. globicipitis* as indicators of migration will increase as additional information about body growth rates at different temperatures is published. Recent growth rate estimations of *X. globicipitis* based on field data^26^ could set temporal limits to migration journeys of Antarctic minke whales based on the observed size of barnacles.

The above inferences are timely, not only for a proper use of the long-term datasets gathered in the history of whaling campaigns [e.g.^33,35,79,83^], but also for future non-lethal research. The ‘tag’ method could be applied to stranded hosts [e.g.^54,57,94,133–136^], but also to data from field surveys given that such ‘tag’ epibionts can be detected visually at moderate distances. There are many examples of photographic records of *P. balaenoptera* and/or coronulids obtained from vessels on both odontocetes [e.g.^25,26,30,137–143^] and baleen whales^53,65,125,144–146^. Even in the case of elusive species, such as the Antarctic minke whale, the advent of novel non-invasive tools (e.g. drone methodology; see e.g.^147^) could assist in collecting future data on epibionts, which are especially relevant in the current climate change scenario.

## Supplementary Information

Supplementary Information.

## Data Availability

The original contributions presented in the study are included in the article/Supplementary Material, further inquiries can be directed to the corresponding author.
