# Supplementary material for: Epibiotic fauna of the Antarctic minke whale as a reliable indicator of seasonal movements
Source: Sci Rep. 2022 Dec 23;12:22214. doi: 10.1038/s41598-022-25929-1 (PMC9789092; doi:10.1038/s41598-022-25929-1)
Supplement: Supplementary file 1 — Supplementary Information. [file 41598_2022_25929_MOESM1_ESM.pdf]

Epibiotic fauna of the Antarctic minke whale as a reliable indicator of seasonal movements

Ten, S., Konishi, K., Raga, J. A., Pastene, L. A., Aznar, F. J.

Supplementary Material

Supplementary figures

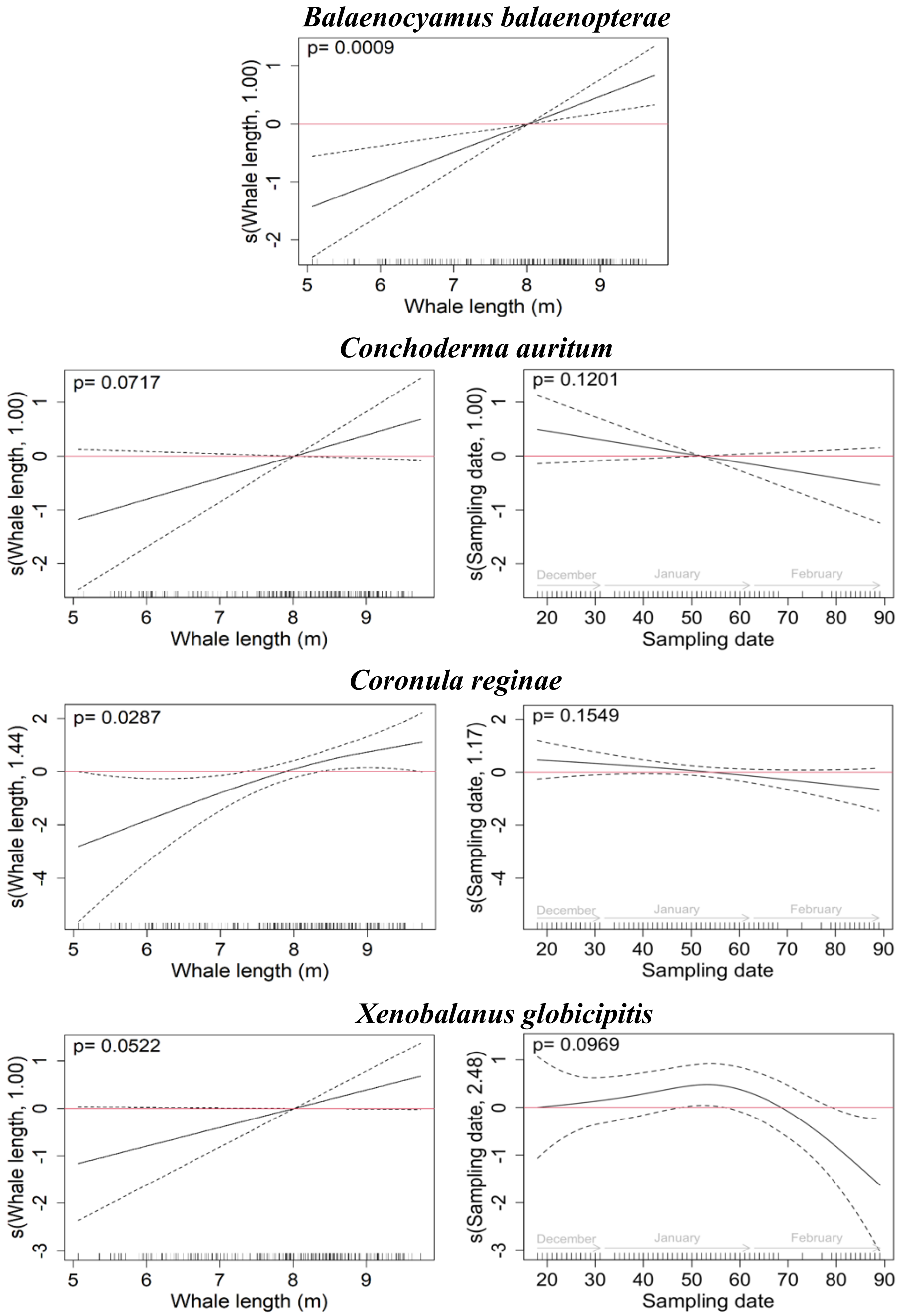

Figure S1. Partial Dependence Plots (PDPs) of the final Generalized Additive Models investigating the influence of whale length and sampling date on the presence of four epibiotic species from Antarctic minke whales, *Balaenoptera bonaerensis*, in the Southern Ocean. The "s" on the y-axis indicates that the variable in brackets is smoothed and the number represents the effective degrees of freedom of the smoothed curves. The ticks on the bottom x-axis (i.e., rug plot) show the number of observations. The dotted lines indicate the 95% confidence intervals, and the red line represents the mean-centered response value.

Supplementary tables

Table S1. Size measurements (mm) of the epibiotic species found on 333 Antarctic minke whales, *Balaenoptera bonaerensis*, captured in the Southern Ocean between December 2018 and February 2019. Values for the total subsample of measured epibionts (n) include the median, the mean, SD (in parentheses), and range [in brackets]; values per whale (N whales) include the average minimum and maximum values, with SD and range. Additional data from voucher specimens from Antarctic minke whales deposited at the Institute for Whale Research of Japan are also included. See text for measurement specifications.

Species	Measured subsample			Measured subsample (per whale)			Voucher specimens	
	n	Median	Mean	N	Minimum	Maximum	n	Mean
Balaenocyamus balaenopterae	-	-	-	-	-	-	-	-
Coronula reginae	54	13.5	14.5 (5.5) [2.9-28.0]	16	12.9 (6.6) [2.9-25.7]	17.0 (6.6) [5.9-28.0]	18	29.3 (13.8) [14.5-58.9]
Coronula diadema	1	8.7	8.7	1	8.7	8.7	-	-
Conchoderma auritum	334	4.7	5.8 (3.5) [0.9-16.8]	18	2.9 (2.4) [0.9-11.7]	11.0 (4.7) [3.0-16.8]	36	4.7 (2.0) [1.3-11.5]
Conchoderma virgatum	1	15.2	15.2	1	15.2	15.2	1	7.4
Xenobalanus globicipitis	89	2.7	3.5 (2.3) [0.5-8.7]	15	2.9 (2.1) [0.5-7.3]	4.5 (2.5) [1.6-8.7]	2	2.8 (0.1) [2.8-2.9]
Pennella balaenoptera	2	59.0	59.0 (28.0) [39.2-78.8]	2	39.2	78.8	1	20.9

Table S2. Studies included in the Generalized Additive Models (GAMs) of the presence/absence of the epibiotic barnacle *Xenobalanus globicipitis* (response variable) as a function of minimum sea temperature (explanatory variable). Cetacean species are arranged in alphabetical order, followed by latitudinal order. SST_{p10} stands for 10th percentile of average annual sea surface temperature (see the text).

Cetacean species	Geographical area	Latitude (º)	Longitude (º)	Year (initial)	Year (final)	SST_{p10} (ºC)	Presence/ absence	Reference
Balaena mysticetus	Alaska (Bering Sea)	71.0	-156,0	1987	1987	-1,8	0	[1]
″	Russia (Sea of Othotsk)	53,0	137,0	2017	2017	-1,8	0	[2]
Balaenoptera edeni	Canary Islands (Atlantic)	27,5	-17,0	1995	2015	19,3	1	[3]
″	Peru (Pacific)	25,0	-109,0	2003	2003	21,1	1	[4]
″	Brazil (Atlantic)	-23,0	-45,0	2020	2020	22,6	1	[5]
″	South Atlantic	-29,0	31,0	1913	1913	20,7	0	[6]
Cephalorhynchus commersonii	Argentina (Atlantic)	-46,0	-65,5	1992	1995	7,8	0	[7]
″	″	-46,0	-65,0	1992	1995	7,6	0	[7]
″	″	-53,5	-65,5	1972	1984	5,6	0	[8]
Delphinapterus leucas	Alaska (Pacific)	60,5	-151,0	2021	2021	-1,1	0	[9]
″	Canada (Atlantic)	48,0	-69,0	1983	2002	-1,8	0	[10]
Delphinus delphis	England (Atlantic)	53,0	-5,0	1990	1994	7,6	0	[11]
″	Belgium (North Sea)	51,0	3,0	1986	1986	3,8	1	[12]
″	Spain (Atlantic)	43,0	-9,5	1991	1996	12,8	0	[13]
″	Spain (Mediterranean)	39,5	0,0	1999	2020	13,8	1	Aznar et al. unpubl.
″	″	39,5	-5,0	1966	1967	15,3	1	[14]
″	Algeria (Mediterranean)	37,0	3,5	1936	1936	14,7	1	[15]
″	US (Pacific)	33,5	-118,5	1970	1976	14,5	1	[16]
″	Mexico (Pacific)	25,0	-109,0	2003	2003	21,1	1	[4]
″	Senegal (Atlantic)	14,0	-17,0	1965	1965	18,3	1	[17]
″	Eastern Tropical Pacific	5,0	-115,0	2003	2003	26,5	1	[4]

″	Brazil (Atlantic)	-25,0	-45,0	2015	2019	21,6	1	[18]
″	South Africa (Atlantic)	-35,0	20,0	1905	1905	15,4	1	[19]
″	New Zealand (Indian)	-36,0	172,0	2007	2014	15,2	0	[20]
″	Argentina (Atlantic)	-40,5	-61,0	1992	1995	9,4	0	[7]
″	Canada (Atlantic)	45,0	-63,0	1966	1966	0,1	1	[21]
″	Spain (Mediterranean)	35,0	-5,0	2001	2015	15,6	1	[22]
Delphinus delphis or *Tursiops truncatus*	Australia (Pacific)	-23,0	151,0	1956	1956	20,4	1	[23]
Feresa attenuata	Japan (Pacific)	35,0	139,0	1994	1994	15,2	0	[24]
″	Mexico (Pacific)	21,0	-90,0	2015	2015	24,1	1	[25]
″	Puerto Rico (Atlantic)	18,0	-67,0	1989	1997	26,3	0	[26]
″	″	18,0	-67,0	1999	1999	26,4	0	[27]
″	Senegal (Atlantic)	14,0	-17,0	1965	1965	18,3	1	[17]
″	Venezuela (Atlantic)	10,0	-64,0	2003	2003	23,3	0	[28]
″	New Caledonia (Pacific)	-21,0	165,0	2014	2014	23,4	0	[29]
″	Australia (Pacific)	-24,0	111,0	1976	1976	21,4	1	[30]
″	Brazil (Atlantic)	-25,0	-45,0	2015	2019	21,6	1	[18]
Globicephala macrorhynchus	Canary Islands (Atlantic)	28,0	-15,0	2012	2012	17,8	1	[31]
″	″	27,5	-17,0	1995	2015	19,3	1	[3]
″	Puerto Rico (Atlantic)	18,2	-66,7	1989	1997	26,2	0	[26]
″	Saint Vincent and the Grenadines (Atlantic)	12,5	-60,5	1971	1971	26,1	1	[32]
″	Eastern Tropical Pacific	5,0	-115,0	2003	2003	26,5	1	[4]
″	Brazil (Atlantic)	-10,0	-32,0	1994	2009	26,3	0	[33]
″	″	-14,0	-39,0	2012	2012	25,5	0	[34]
″	″	-25,0	-45,0	2015	2019	21,6	1	[18]
Globicephala melas	Faroe Islands (Norwegian Sea)	61,5	-6,5	1987	1988	6,7	1	[35, 36]
″	″	61,5	-6,5	1883	1883	6,6	1	[37]
″	″	61,5	-6,5	1921	1921	6,6	1	[38]

″	″	61,5	-6,5	1978	1978	6,6	1	[39]
″	″	61,5	-6,5	1988	1988	6,6	1	[40]
″	″	61,5	-6,5	1851	1851	6,6	1	[41]
″	″	61,5	-6,5	1897	1897	6,6	1	[42]
″	England (Atlantic)	53,0	-5,0	1990	1994	7,6	0	[11]
″	France (Atlantic)	47,0	-2,6	1987	1988	9,3	1	[36]
″	Spain (Atlantic)	43,0	-8,0	1991	1996	12,9	0	[13]
″	Monaco (Ligurian Sea)	43,0	7,0	1912	1912	12,9	1	[43]
″	US (Atlantic)	41,0	-69,0	1963	1963	5,2	1	[44]
″	Spain (Mediterranean)	39,5	0,0	1993	2003	13,8	1	[36, 45], Aznar et al. unpubl.
″	″	39,5	2,5	1936	1936	13,9	1	[15]
″	″	39,0	0,0	1920	1920	13,5	1	[46]
″	″	39,0	0,0	1969	1969	13,5	1	[47]
″	US (Atlantic)	36,0	-71,0	1916	1916	19,3	1	[48]
″	Spain (Mediterranean)	35,0	-5,0	2001	2015	15,6	1	[22]
″	South Shetlands (Antarctica)	-62,0	-59,0	1913	1913	-1,5	1	[49]
Grampus griseus	Italy (Mediterranean)	40,5	13,5	2000	2000	14,0	0	[50]
″	Spain (Mediterranean)	39,9	0,6	2021	2021	13,5	1	Aznar et al. unpubl.
″	″	39,5	0,0	1999	2020	13,8	1	Aznar et al. unpubl.
″	Azores (Atlantic)	38,0	-28,0	1920	1920	15,4	1	[46]
″	Eastern Tropical Pacific	5,0	-115,0	2003	2003	26,5	1	[4]
″	Brazil (Atlantic)	-25,0	-45,0	2015	2019	21,6	1	[18]
″	South Africa (Atlantic)	-35,0	20,0	1984	1984	15,3	1	[51]
Kogia breviceps	Canada (Atlantic)	45,0	-66,0	1992	1992	1,5	0	[52]
″	US (Atlantic)	37,0	-66,0	1941	1941	19,4	0	[53]
″	Puerto Rico (Atlantic)	18,0	-65,0	1989	1997	26,0	0	[26]
″	Brazil (Atlantic)	-10,0	-32,0	1994	2009	26,3	0	[33]

Kogia sima	″	-10,0	-32,0	1994	2009	26,3	0	[33]
Kogia sp.	US (Atlantic)	27,0	-97,0	1973	1973	18,4	1	[54]
Leucopleurus acutus	England (Atlantic)	53,0	-5,0	1990	1994	7,6	0	[11]
″	North Sea	53,0	5,5	1990	2019	4,2	0	[55]
Lagenorhynchus albirostris	Netherlands (North Sea)	53,0	6,0	1978	1978	4,2	0	[56]
″	England (Atlantic)	53,0	-5,0	1990	1994	7,6	0	[11]
″	North Sea	53,0	5,5	2008	2019	4,1	0	[55]
Sagmatias obliquidens	US (Pacific)	33,5	-118,5	1970	1976	14,5	1	[16]
Sagmatias obscurus	Namibia (Atlantic)	-18,0	11,0	2002	2002	14,3	1	[57, 58]
″	Peru (Pacific)	-12,0	-77,0	1985	1990	15,7	1	[59]
″	Argentina (Atlantic)	-40,5	-61,0	1990	1995	9,7	0	[60]
Lissodelphis borealis	US (Pacific)	33,5	-118,5	1970	1976	14,5	1	[16]
Mesoplodon bidens	Canada (Atlantic)	51,0	-55,0	1957	1957	-1,8	0	[61]
″	Canary Islands (Atlantic)	28,0	-13,0	2011	2011	18,0	1	[62]
Mesoplodon carlhubbsi	Alaska (Bering Sea)	51,0	-176,0	1999	1999	3,1	0	[63]
″	US (Pacific)	37,0	-124,0	1989	1989	11,5	0	[64]
Mesoplodon densirostris	Chile (Pacific)	-53,0	-75,0	1990	1990	6,9	0	[65]
Mesoplodon europaeus	Brazil (Atlantic)	12,0	-69,0	1992	1992	25,7	0	[66]
″	US (Atlantic)	34,5	-76,0	1989	1989	20,7	0	[64]
Mesoplodon hectori	US (Pacific)	37,0	-124,0	1989	1989	11,5	0	[64]
Mesoplodon layardii	South Africa (Atlantic)	-32,0	18,0	1996	1996	13,0	0	[67]
″	Australia (Pacific)	-33,0	151,0	1963	1963	17,5	0	[68]
″	″	-38,0	144,0	1980	1980	12,4	0	[69]
″	Argentina (Atlantic)	-48,0	-64,0	1992	1992	6,1	0	[70]
Mesoplodon mirus	US (Atlantic)	38,5	-74,0	1989	1989	7,7	1	[64]
″	Brazil (Atlantic)	-23,0	-45,0	2005	2005	22,3	0	[71]
″	Mozambique (Indian)	-24,0	33,5	2016	2016	22,7	0	[72]

″	South Africa (Atlantic)	-35,0	20,0	1984	1984	15,3	1	[51]
Mesoplodon stejnegeri	Alaska (Bering Sea)	51,0	-176,0	2021	2021	3,7	0	[73]
″	Japan (Pacific)	39,0	134,5	1999	2011	8,5	0	[74]
Monodon monoceros	Greenland (Baffin Bay)	69,0	-56,0	1922	1922	-1,8	0	[75]
Neophocaena asiaeorientalis	China (Yellow Sea)	39,5	120,0	2015	2016	1,5	0	[76]
″	Korea (East Sea)	34,0	128,0	2016	2016	14,0	1	[77]
″	Japan (Pacific)	34,0	134,5	2017	2017	14,4	0	[78]
Neophocaena phocaenoides	China (China Sea)	23,5	118,0	2000	2000	16,7	0	[79]
″	China (South China Sea)	22,0	113,5	1993	1998	19,0	1	[80]
″	India (Arabian Sea)	14,0	74,0	1965	1965	26,9	1	[81]
Peponocephala electra	US (Atlantic)	27,0	-80,0	2006	2006	23,7	0	[82]
″	Cape Verde (Atlantic)	15,5	-22,0	2008	2008	22,2	0	[83]
″	Brazil (Atlantic)	-10,0	-32,0	1994	2009	26,3	0	[33]
Phocoena phocoena	Iceland (Atlantic)	65,5	-21,5	2000	2000	0,0	0	[84]
″	Norway (Norway Sea)	64,0	14,0	2000	2000	4,4	0	[84]
″	Baltic Sea	57,5	19,0	1990	2015	2,4	0	[85]
″	Baltic Sea	55,0	16,5	1990	2015	2,7	0	[85]
″	Baltic Sea	54,0	7,5	2006	2018	4,3	0	[86]
″	Baltic Sea	54,0	12,5	1990	2015	2,3	0	[85]
″	England (Atlantic)	53,0	-5,0	1990	1994	7,6	0	[11]
″	US (Pacific)	44,0	-125,0	1973	1977	9,8	0	[87]
″	Spain (Atlantic)	42,5	-8,0	1991	1996	13,0	0	[13]
″	Greece (Aegean Sea)	36,0	25,0	2013	2013	16,3	0	[88]
″	Spain (Alboran Sea)	35,5	-3,0	2006	2006	15,2	1	[89]
″	Senegal (Atlantic)	14,0	-17,0	1965	1965	18,3	1	[17]
Phocoena sinus	Mexico (Pacific)	30,5	-114,0	1999	1999	16,4	1	[90]
″	″	31,6	-114,7	1985	1985	14,9	1	[91]
Phocoena spinnipinnis	Peru (Pacific)	-19,0	-78,0	1983	1989	17,2	1	[92]
Phocoenoides dalli	US (Pacific)	33,5	-118,5	1970	1976	14,5	0	[16]

Physeter macrocephalus	Arctic	83,0	60,0	1925	1925	-1,8	0	[93]
″	Russia (Bering Sea)	55,0	166,0	1972	1972	1,0	0	[94]
″	″	54,5	166,5	1957	1957	1,4	0	[95]
″	Ireland (Atlantic)	54,0	-10,0	1914	1914	8,5	0	[96]
″	″	54,0	-10,0	1909	1909	8,5	0	[97]
″	″	54,0	-10,0	2003	2003	8,7	0	[98]
″	Alaska (Bering Sea)	54,0	-166,0	1938	1938	3,5	0	[99]
″	Canada (Pacific)	53,0	-131,5	1950	1950	6,9	0	[100]
″	North Sea	53,0	4,5	2018	2018	5,2	0	[101]
″	France (Mediterranean)	43,0	3,0	1993	1993	11,9	0	[102]
″	Japan (Pacific)	42,0	144,0	1935	1935	1,1	0	[103]
″	Azores (Atlantic)	38,0	-28,0	1955	1955	15,4	0	[104]
″	″	38,0	-28,0	1949	1954	15,4	0	[105]
″	Japan (Pacific)	38,0	143,0	1958	1958	9,3	0	[106]
″	Atlantic	38,0	-28,0	1902	1902	15,4	0	[107]
″	US (Pacific)	37,5	-124,0	1959	1962	11,5	0	[108]
″	Spain (Mediterranean)	35,0	-5,0	2001	2015	15,6	0	[22]
″	Japan (Pacific)	35,0	137,5	1946	1949	14,4	0	[109]
″	″	26,0	142,0	1950	1950	20,8	0	[110]
″	Puerto Rico (Atlantic)	18,0	-67,0	1989	1997	26,3	0	[26]
″	Brazil (Atlantic)	-17,0	-39,0	1993	1993	25,0	1	[111]
″	South Africa (Indian)	-29,0	31,0	1926	1931	20,7	0	[112]
″	South Africa (Atlantic)	-33,0	18,0	1963	1963	14,0	0	[113]
″	″	-35,0	20,0	1939	1939	15,4	0	[114]
″	New Zealand (Pacific)	-38,0	171,0	1874	1874	14,4	0	[115]
″	South Georgia and the South Sandwich Islands (Atlantic)	-56,0	-31,0	1927	1931	-1,2	0	[112]
″	South Shetland Islands (Antarctica)	-62,0	-59,0	1930	1930	-1,5	0	[116]
″	″	-62,0	-59,0	1930	1930	-1,5	0	[117]
″	Antarctica	-63,0	-36,0	1950	1951	-1,8	0	[118]
″	″	-69,0	-171,0	1948	1952	-1,8	0	[119]

″	″	-69,0	-170,0	1951	1952	-1,8	0	[120]
″	″	-76,0	-42,0	1950	1950	-1,8	0	[121]
″	″	-76,0	-42,0	1951	1951	-1,8	0	[122]
″	″	-76,0	-42,0	1971	1971	-1,8	0	[123]
″	″	-76,0	-42,0	1962	1962	-1,8	0	[124]
Pontoporia blainvillei	Brazil (Atlantic)	-21,5	-39,0	1986	1999	23,5	1	[125, 126]
″	″	-30,5	-50,0	1992	1995	15,3	0	[127]
″	Uruguay (Atlantic)	-34,0	-53,0	1971	1973	12,3	1	[128]
″	″	-34,0	-52,0	2002	2002	13,4	1	[129]
″	″	-34,0	-52,0	1989	1989	14,0	1	[130]
″	Argentina (Atlantic)	-35,0	-56,5	2009	2012	10,9	0	[131]
Pseudorca crassidens	Monaco (Ligurian Sea)	43,0	7,0	1912	1912	12,9	1	[43]
″	Spain (Mediterranean)	39,0	0,0	1967	1967	13,5	1	[132]
″	Azores (Atlantic)	38,0	-28,0	1920	1920	15,4	1	[46]
″	″	38,0	-28,0	1936	1936	15,4	1	[15]
″	Brazil (Atlantic)	-25,0	-45,0	2015	2019	21,6	1	[18]
Sotalia fluviatilis	″	-21,5	-39,0	1987	2002	23,6	1	[133]
Sotalia guianensis	″	-10,0	-32,0	1994	2009	26,3	0	[33]
″	″	-22,0	-43,0	2021	2021	21,7	1	[134]
″	″	-23,0	-45,0	2020	2020	22,6	1	[5]
″	″	-23,8	-45,6	1991	1991	20,1	1	[135]
Sousa chinensis	South China Sea	22,0	113,5	1993	1998	19,0	0	[80]
″	South Africa (Atlantic)	-35,0	20,0	1994	1994	15,4	0	[136]
Sousa plumbea	Greece (Mediterranean)	35,0	25,0	2018	2018	16,7	0	[137]
″	South Africa (Indian)	-29,0	32,0	2010	2012	21,4	1	[138]
Stenella attenuata	Japan (Pacific)	33,0	135,0	1979	1979	18,2	1	[139]
″	Eastern Tropical Pacific	5,0	-115,0	2003	2003	26,5	1	[4]
″	Mozambique (Indian)	-18,0	38,0	1984	1984	24,3	1	[51]
″	Brazil (Atlantic)	-25,0	-45,0	2015	2019	21,6	1	[18]
Stenella clymene	US (Gulf of Mexico)	29,0	-94,0	1981	1992	16,7	1	[140]
″	Brazil (Atlantic)	-10,0	-32,0	1994	2009	26,3	0	[33]

Stenella coeruleoalba	England (Atlantic)	53,0	-5,0	1990	1994	7,6	0	[11]
″	Galicia (Atlantic)	43,0	-9,5	1991	1996	12,8	0	[13]
″	Italy (Mediterranean)	41,5	12,0	1985	1990	13,8	0	[141]
″	Spain (Mediterranean)	41,0	2,0	1997	1997	13,1	1	[142]
″	Italy (Mediterranean)	40,5	13,5	2000	2000	14,0	0	[50]
″	Spain (Mediterranean)	39,5	2,5	1979	1979	13,9	0	[139]
″	″	39,5	0,0	1983	2021	13,6	1	[143-147]
″	″	39,5	2,5	1988	1988	14,4	1	[40]
″	″	38,0	0,0	1992	1992	14,0	1	[148]
″	Italy (Mediterranean)	37,0	14,0	2014	2014	15,3	0	[149]
″	″	37,0	15,0	1998	1998	14,9	1	[150]
″	Spain (Mediterranean)	36,0	-5,5	1966	1967	15,3	1	[14]
″	Costa Rica (Pacific)	9,0	-84,0	2001	2009	27,9	1	[151]
″	Eastern Tropical Pacific	5,0	-115,0	2003	2003	26,5	1	[4]
″	Brazil (Atlantic)	-4,0	-37,0	2007	2007	26,1	1	[152]
″	South Africa (Atlantic)	-35,0	20,0	1984	1984	15,3	1	[51]
″	New Zealand (Pacific)	-38,5	173,5	2007	2014	14,3	0	[20]
Stenella frontalis	US (Atlantic)	35,4	-75,5	2017	2017	14,9	1	[153]
″	Canary Islands (Atlantic)	27,5	-17,0	1995	2015	19,3	1	[3]
″	Puerto Rico (Atlantic)	18,0	-65,0	1989	1997	26,0	0	[26]
″	Brazil (Atlantic)	-23,0	-45,0	2020	2020	22,6	1	[5]
Stenella longirostris	Eastern Tropical Pacific	21,0	-108,0	2003	2003	23,8	1	[4]
″	″	9,0	-145,0	2003	2003	26,7	1	[4]
″	″	-5,0	-98,0	2003	2003	22,1	1	[4]
″	Brazil (Atlantic)	-10,0	-32,0	1994	2009	26,3	0	[33]
″	″	-25,0	-45,0	2015	2019	21,6	1	[18]
Steno bredanensis	Canary Islands (Atlantic)	27,5	-17,0	1995	2015	19,3	1	[3]
″	Mauretania (Atlantic)	20,0	-17,0	1988	1988	17,5	1	[154]
″	Brazil (Atlantic)	-3,0	-38,0	2020	2020	26,7	1	[155]
″	″	-10,0	-32,0	1994	2009	26,3	0	[33]
″	″	-23,0	-45,0	2020	2020	22,6	1	[5]
Tursiops aduncus	″	-5,0	-35,0	1919	1919	26,2	1	[156]

″	South Africa (Indian)	-33,5	25,0	1984	1984	16,6	1	[157]
″	Japan (Pacific)	38,0	143,0	2006	2006	9,6	1	[158]
Tursiops truncatus	Arctic	83,0	60,0	1925	1925	-1,8	0	[93]
″	England (Atlantic)	53,0	-5,0	1990	1994	7,6	0	[11]
″	Spain (Atlantic)	43,0	-8,5	1991	1996	12,9	0	[13]
″	Italy (Mediterranean)	40,5	13,5	2000	2000	14,0	0	[50]
″	Spain (Mediterranean)	39,5	0,0	1999	2020	13,8	1	Aznar et al. unpubl.
″	US (Atlantic)	39,0	-74,0	2005	2005	5,0	1	[159]
″	″	39,0	-74,0	2003	2005	5,0	1	[160]
″	Turkey (Aegean Sea)	37,0	27,0	2017	2017	16,3	0	[161]
″	Morocco (Mediterranean)	35,0	-5,0	1966	1966	15,4	1	[162]
″	Spain (Mediterranean)	35,0	-5,0	2001	2015	15,6	1	[22]
″	US (Atlantic)	35,0	-75,0	1886	1886	21,3	1	[163]
″	US (Pacific)	35,0	-74,0	2003	2012	21,8	1	[164]
″	US (Atlantic)	34,5	-76,5	1990	1990	18,2	1	[165]
″	″	34,0	-76,0	1993	2003	22,2	1	[166]
″	″	34,0	-78,0	2014	2014	13,4	1	[167]
″	US (Pacific)	33,5	-118,5	1997	2007	14,3	1	[168]
″	Japan (Pacific)	33,0	135,0	1979	1979	18,2	0	[139]
″	US (Atlantic)	29,2	-94,9	1990	1992	15,7	1	[169]
″	US (Gulf of Mexico)	29,0	-94,0	2005	2005	17,0	1	[170]
″	US (Atlantic)	29,0	-95,0	1994	1994	14,9	1	[171]
″	Canary Islands (Atlantic)	28,0	-17,0	1992	1992	18,8	0	[172]
″	″	27,5	-17,0	1995	2015	19,3	1	[3]
″	US (Atlantic)	27,0	-80,5	2001	2001	22,1	1	[173]
″	Mexico (Gulf of Mexico)	25,5	-97,0	2014	2016	19,6	1	[174]
″	″	25,0	-94,0	1986	1986	22,4	1	[175]
″	Puerto Rico (Atlantic)	18,0	-65,0	1989	1997	26,0	0	[26]
″	India (Bay of Bengal)	11,0	79,0	1998	1998	27,6	1	[176]
″	″	11,0	79,0	1985	1985	26,5	1	[177]
″	Costa Rica (Pacific)	8,0	-83,0	2020	2020	27,7	1	[178]

″	Eastern Tropical Pacific	5,0	-115,0	2003	2003	26,5	1	[4]
″	Peru (Pacific)	-12,0	-76,0	1990	1990	16,0	1	[179]
″	Angola (Atlantic)	-15,0	12,0	2008	2008	18,7	1	[180]
″	Mexico (Gulf of Mexico)	-21,0	-97,0	2007	2007	19,7	1	[181]
″	Brazil (Atlantic)	-21,5	-39,0	1989	1998	23,6	1	[125]
″	″	-23,0	-43,0	1996	1996	20,2	1	[182]
″	″	-23,0	-45,0	2020	2020	22,6	1	[5]
″	Australia (Coral Sea)	-27,0	153,0	1993	1994	20,2	1	[183]
″	Chile (Pacific)	-33,0	-71,0	2005	2007	11,8	1	[184]
″	South Africa (Atlantic)	-35,0	20,0	1984	1984	15,3	1	[51]
″	Argentina (Atlantic)	-41,5	64,5	1999	2012	13,0	0	[185]
Ziphius cavirostris	Central Mediterranean	43,5	8,5	1998	2007	13,1	1	[186]
″	Spain (Mediterranean)	39,5	0,0	1999	2020	13,8	1	Aznar et al. unpubl.
″	Algeria (Mediterranean)	37,0	3,5	1938	1938	14,7	0	[187]
″	US (Atlantic)	34,0	-77,0	1978	1978	18,4	1	[188]
″	Canary Islands (Atlantic)	27,5	-17,0	1995	2015	19,3	1	[3]
″	Puerto Rico (Atlantic)	17,0	-64,5	1989	1997	26,2	0	[26]
″	Ecuador (Pacific)	0,0	-90,0	1923	2003	21,7	1	[189]
″	Indonesia (Banda Sea)	-3,0	128,0	1976	1976	27,6	0	[190]
″	New Zealand (Pacific)	-43,0	172,0	1978	1978	9,1	0	[191]
″	Croatia (Mediterranean)	42,0	18,0	2005	2005	14,2	0	[192]

Table S3. Data on the presence (or absence) of the epibiotic barnacle *Xenobalanus globicipitis* on cetaceans typically performing long latitudinal migrations. Cetacean species are arranged in alphabetical order, followed by the variable region (‘1’: sampled within 50ºN-40ºS, ‘2’: outside this range; see the text).

Cetacean species	Geographical area	Region	Presence/ absence	Reference
Balaenoptera acutorostrata	Japan (Pacific)	1	1	[193]
″	″	1	1	[194]
″	″	1	1	[195]
″	″	1	1	[196]
″	Strait of Gibraltar	1	0	[22]
″	Turkey (Mediterranean)	1	0	[197]
″	US (Pacific)	1	0	[198]
″	″	1	1	[199]
″	Iceland (Atlantic)	2	1	[200]
″	″	2	1	[201]
Balaenoptera bonaerensis	Antarctica	2	0	[202]
″	″	2	0	[203]
″	″	2	0	[204]
″	″	2	0	[49]
″	″	2	0	[205]
″	″	2	0	[206]
″	″	2	1	[207]
″	″	2	1	This study
Balaenoptera borealis	Canada (Pacific)	1	1	[208]
″	Japan (Pacific)	1	0	[109]
″	South Africa	1	1	[209]
″	South Africa (Atlantic)	1	1	[209]
″	″	1	1	[116]
″	Tunisia (Mediterranean)	1	1	[210]
″	US (Atlantic)	1	0	[211]

″	US (Pacific)	1	1	[212]
″	″	1	1	[108]
″	″	1	1	[213]
″	Antarctica	2	0	[203]
″	Barents Sea	2	0	[214]
″	Faroe Islands (Norwegian Sea)	2	1	[215]
″	Greenland	2	1	[215]
″	Kerguelen Islands (Indian)	2	1	[216]
″	″	2	1	[216]
″	″	2	1	[216]
″	North Sea, Norwegian Sea	2	0	[39]
″	Norway (Barents Sea)	2	1	[215]
″	″	2	1	[38]
″	″	2	1	[40]
″	Norway (Norwegian Sea)	2	1	[217]
″	South Shetland Islands (Antarctica)	2	0	[218]
″	″	2	0	[49]
″	″	2	0	[219]
Balaenoptera musculus	Brazil (Atlantic)	1	0	[220]
″	Canada (Pacific)	1	1	[221]
″	Canada/US (Pacific)	1	1	[222]
″	Eastern Tropical Pacific	1	1	[4]
″	Ireland (Atlantic)	1	0	[97]
″	″	1	0	[223]
″	Japan (Pacific)	1	0	[109]
″	South Africa	1	0	[224]
″	″	1	1	[225]
″	South Africa (Atlantic)	1	1	[156]
″	″	1	1	[116]
″	″	1	1	[114]
″	UK (North Sea)	1	1	[40]
″	US (Pacific)	1	1	[108]

″	″	1	1	[213]
″	Antarctica	2	0	[119]
″	″	2	0	[226]
″	″	2	0	[227]
″	″	2	0	[120]
″	″	2	0	[122]
″	″	2	0	[123]
″	″	2	0	[228]
″	″	2	0	[229]
″	″	2	0	[118]
″	″	2	0	[95]
″	Barents Sea	2	0	[230]
″	Norway (Norwegian Sea)	2	0	[217]
″	″	2	0	[231]
″	South Georgia and the South Sandwich Islands (Atlantic)	2	0	[232]
″	″	2	1	[225]
″	South Shetland Islands (Antarctica)	2	0	[49]
Balaenoptera physalus	Canada (Atlantic)	1	1	[233]
″	Eastern Tropical Pacific	1	1	[4]
″	Italy (Mediterranean)	1	0	[234]
″	Japan (Pacific)	1	0	[109]
″	Madeira (Atlantic)	1	0	[235]
″	Mediterranean	1	0	[236]
″	South Africa	1	1	[156]
″	″	1	1	[225]
″	South Africa (Atlantic)	1	1	[116]
″	Spain (Atlantic)	1	1	[237]
″	″	1	1	[238]
″	Strait of Gibraltar	1	0	[22]
″	Tunisia (Mediterranean)	1	1	[210]
″	″	1	1	[150]

″	US (Pacific)	1	0	[208]
″	″	1	0	[108]
″	Antarctica	2	0	[120]
″	″	2	0	[216]
″	″	2	0	[122]
″	″	2	0	[123]
″	″	2	0	[228]
″	″	2	0	[229]
″	″	2	0	[118]
″	″	2	1	[239]
″	Denmark (North Sea)	2	0	[39]
″	Faroe Islands (Norwegian Sea)	2	0	[240]
″	Iceland (Atlantic)	2	0	[241]
″	Norway (Norwegian Sea)	2	0	[231]
″	South Georgia and the South Sandwich Islands (Atlantic)	2	0	[242]
″	″	2	1	[225]
″	South Shetland Islands (Antarctica)	2	0	[49]
″	″	2	0	[114]
″	″	2	1	[116]
Berardius bairdii	Japan (Pacific)	1	0	[243]
″	″	1	0	[109]
″	US (Pacific)	1	0	[108]
Eschrichtius robustus	Canada/US (Pacific)	1	0	[244]
″	″	1	0	[245]
″	Japan (Pacific)	1	0	[246]
″	Mexico (Pacific)	1	0	[247]
″	″	1	0	[48]
″	US (Pacific)	1	0	[248]
″	″	1	0	[108]
″	″	1	0	[249]
″	″	1	0	[250]
″	″	1	0	[251]

″	″	1	0	[252]
Eubalaena australis	South Africa	1	0	[253]
″	South Africa (Atlantic)	1	0	[254]
Eubalaena glacialis	Canada (Atlantic)	1	0	[255]
″	Alaska (Bering Sea)	2	0	[99]
″	Norway (Norwegian Sea)	2	0	[217]
Eubalaena japonica	Japan (Pacific)	1	0	[256]
Hyperodoon ampullatus	Arctic	2	0	[93]
″	″	2	0	[257]
″	Norwegian Sea	2	0	[215]
″	Ireland (Atlantic)	1	0	[258]
″	UK (North Sea)	1	0	[259]
″	Faroe Islands (Norwegian Sea)	2	0	[39]
″	Norway (Norwegian Sea)	2	0	[217]
″	South Shetland Islands (Antarctica)	2	0	[49]
Hyperoodon planifrons	″	2	0	[49]
Megaptera novaeangliae	Arabian Sea	1	1	[260]
″	Argentina (Atlantic)	1	0	[261]
″	″	1	0	[262]
″	Brazil (Atlantic)	1	0	[33]
″	″	1	0	[263]
″	″	1	0	[135]
″	Canada (Pacific)	1	0	[264]
″	″	1	0	[208]
″	″	1	0	[48]
″	Colombia (Pacific)	1	0	[265]
″	Colombia (Pacific)	1	0	[266]
″	Hawaii (Pacific)	1	0	[267]
″	Ireland (Atlantic)	1	0	[268]
″	Japan (Pacific)	1	0	[103]
″	″	1	0	[269]

″	″	1	0	[109]
″	Madagascar (Indian)	1	0	[270]
″	New Caledonia (Pacific)	1	0	[271]
″	New Zealand (Pacific)	1	0	[272]
″	″	1	0	[273]
″	Peru (Pacific)	1	1	[4]
″	Puerto Rico (Atlantic)	1	0	[26]
″	South Africa	1	0	[274]
″	South Africa (Indian)	1	0	[253]
″	Spain (Atlantic)	1	0	[13]
″	US (Pacific)	1	0	[212]
″	″	1	0	[275]
″	″	1	0	[108]
″	″	1	0	[250]
″	Alaska (Bering Sea)	2	0	[99]
″	Antarctica	2	0	[119]
″	″	2	0	[276]
″	″	2	0	[277]
″	″	2	0	[120]
″	″	2	0	[122]
″	″	2	0	[123]
″	″	2	0	[118]
″	″	2	0	[278]
″	Faroe Islands (Norwegian Sea)	2	0	[215]
″	Greenland	2	0	[279]
″	Iceland (Atlantic)	2	0	[241]
″	Kerguelen Islands (Indian)	2	0	[280]
″	Netherlands (North Sea)	2	0	[281]
″	Norway (Barents Sea)	2	0	[39]
″	Norway (Norwegian Sea)	2	0	[217]
″	″	2	0	[219]
″	″	2	0	[282]

″	″	2	0	[283]
″	South Georgia and the South Sandwich Islands (Atlantic)	2	0	[242]
″	″	2	0	[117]
″	South Shetland Islands (Antarctica)	2	0	[218]
″	″	2	0	[49]
″	″	2	0	[114]
Orcinus orca	Australia (Pacific)	1	1	[284]
″	Brazil (Atlantic)	1	0	[285]
″	″	1	1	[5]
″	Canary Islands (Atlantic)	1	1	[286]
″	Chile (Pacific)	1	1	[287]
″	Costa Rica (Pacific)	1	1	[288]
″	″	1	1	[289]
″	Ecuador (Pacific)	1	1	[290]
″	Japan (Pacific)	1	1	[291]
″	Mexico (Pacific)	1	1	[292]
″	″	1	1	[293]
″	Monaco (Ligurian Sea)	1	1	[294]
″	New Zealand (Pacific)	1	1	[295]
″	Peru (Pacific)	1	1	[296]
″	South Africa	1	1	[297]
″	″	1	1	[298]
″	″	1	1	[298]
″	Spain/France (Mediterranean)	1	1	[46]
″	Strait of Gibraltar	1	1	[22]
″	″	1	1	[15]
″	US (Pacific)	1	1	[299]
″	Canada (Atlantic)	2	1	[300]

References

1. Heckmann, R. A., Jensen, L. A., Warnock, R. G., & Coleman, B. Parasites of the bowhead whale, *Balaena mysticetus*. *Great Basin Nat.* 355-372 (1987).
2. Chernova, O. F., Shpak, O. V., Kiladze, A. B., & Rozhnov, V. V. Epidermal Molting in the Bowhead Whale *Balaena mysticetus*. *Biol. Bull. Russ. Acad. Sci.* **44**, 591-602 (2017).
3. Kautek, G., Van Bressem, M. F., & Ritter, F. External body conditions in cetaceans from La Gomera, Canary Islands, Spain. *J. Mar. Anim. Ecol.* **11**, 4-17 (2008).
4. Kane, E. A., Olson, P. A., Gerodette, T., & Fiedler, P. C. Prevalence of the commensal barnacle *Xenobalanus globicipitis* on cetacean species in the eastern tropical Pacific Ocean, and review of global occurrence. *Fish. Bull.* **106**, 395-404 (2008).
5. Siciliano S., Cardoso J., Francisco A., De Souza S. P., Hauser-Davis R. A., & Iwasa-Arai T. Epizoic barnacle (*Xenobalanus globicipitis*) infestations in several cetacean species in south-eastern Brazil. *Mar. Biol. Res.* **16**, 1-13 (2020).
6. Olsen, O. On the external characters and biology of Bryde's whales (*Balaenoptera brydei*), a new rorqual from the coast of South Africa. *Proc. Zool. Soc. Lond.* 1073-1090 (1913).
7. Berón-Vera, B., Pedraza, S. N., Raga, J. A., de Pertierra, A. G., Crespo, E. A., Alonso, M. K., & Goodall, R. N. P. Gastrointestinal helminths of Commerson's dolphins *Cephalorhynchus commersonii* from central Patagonia and Tierra del Fuego. *Dis. Aquat. Org.* **47**, 201-208 (2001).
8. Goodall, R. N. P., Galeazzi, A. R., Leatherwood, S., Miller, K. W., Cameron, I. S., Kastelein, R. K., & Sobral, A. P. Studies of Commerson's dolphins, *Cephalorhynchus commersonii*, off Tierra del Fuego, 1976–1984. *Rep. Int. Whal. Commn* **9**, 143-160 (1988).
9. Burek-Huntington, K. Personal communication (2021).
10. Lair, S., Measures, L. N., & Martineau, D. Pathologic findings and trends in mortality in the beluga (*Delphinapterus leucas*) population of the St Lawrence Estuary, Quebec, Canada, from 1983 to 2012. *Vet. Pathol.* **53**, 22-36 (2016).
11. Gibson, D. I., Harris, E. A., Bray, R. A., Jepson, P. D., Kuiken, T., Baker, J. R., *et al.* A survey of the helminth parasites of cetaceans stranded on the coast of England and Wales during the period 1990-1994. *J. Zool.* **244**, 563-574 (1998).
12. Rappé, G. Een vondst van *Xenobalanus globicipitis* in de Noordzee? *Strandvlo* **8**, 100-101 (1988).
13. Abollo, E., López, A., Gestal, C., Benavente, P., & Pascual, S. Macroparasites in cetaceans stranded on the northwestern Spanish Atlantic coast. *Dis. Aquat. Org.* **32**, 227-231 (1998).
14. Pilleri, G. *Xenobalanus globicipitis* Steenstrup on *Delphinus delphus*, *Stenella styx, Tursiops truncatus* in the western Mediterranean. *Invest. Cet.* **2**, 248-249 (1970).
15. Richard, J. Résultats des campagnes scientifiques accomplies sur son yacht par Albert 1er Prince Souverain de Monaco. *Fascicule XCIV* **94**, 34-71 (1936).
16. Dailey, M. D., & Walker, W. A. Parasitism as a Factor (?) in Single Strandings of Southern California Cetaceans. *J. Parasitol.* **64**, 593-596; 10.2307/3279939 (1978).
17. Stubbings, H. G. West African Cirripedia in the collection of the Institut Français d'Afrique Noire, Dakar, Senegal. *Bull. l'Institut Fran. d'Afrique Noire* **27**, 876-907 (1965).
18. Minussi Rama, A. C. Prevalência, intensidade e distribuição de *Xenobalanus Globicipitis* (Cirripedia: Coronulidae) em cetáceos na Bacia de Santos, Brasil. [Bachelor Thesis] (2020).
19. Gruvel, J. A. Monographie des Cirrhipèdes ou Thécostracés. *Paris: Masson et cie* (1905).
20. Lehnert, K., Randhawa, H., & Poulin, R. Metazoan parasites from odontocetes off New Zealand: new records. *Parasitol. Res.* **116**, 2861-2868 (2017).

21. Blum, S., & Fong, J. CAS Invertebrate Zoology (IZ). Version 14.2. *California Academy of Sciences* https://www.gbif.org/occurrence/609385701 [Accessed October 6, 2021] (2016).
22. Herr, H., Burkhardt-Holm, P., Heyer, K., Siebert, U., & Selling, J. Injuries, Malformations, and Epidermal Conditions in Cetaceans of the Strait of Gibraltar. *Aquat. Mamm.* **46**, 215-235 (2020).
23. Pope, E. C. The barnacle, *Xenobalanus globicipitis* Steenstrup in Australian Seas. *Proc. R. Zool. Soc.* 159-161 (1958).
24. Terasawa, F., Yamagami, T., Kitamura, M., & Fujimoto, A. A pygmy killer whale (*Feresa attenuata*) stranded at Sagami Bay, Japan. *Aquat. Mamm.* **23**, 69-72 (1997).
25. Díaz-Gamboa, R. E. Varamiento de orcas pigmeas (*Feresa attenuata* Gray 1874) en Yucatán: reporte de caso. *Bioagrociencias* **8**, 36-43 (2015).
26. Mignucci-Giannoni, A. A., Hoberg, E. P., Siegel-Causey, D., & Williams Jr, E. H. Metazoan parasites and other symbionts of cetaceans in the Caribbean. *J. Parasitol.* 939-946 (1998).
27. Rodríguez-López, M. A., and Mignucci-Giannoni, A. A. A stranded pygmy killer whale (*Feresa attenuata*) in Puerto Rico. *Aquat. Mamm.* **25**, 119-121 (1999).
28. Bermúdez-Villapol, L. A., Sayegh, A. J., Estevez, M. A., Rangel, M. S., Rosso, C., & Vera, N. I. Notes on the pygmy killer whale *Feresa attenuata* Gray, 1874 (Cetacea: Delphinidae) in Venezuela, southeastern Caribbean. *Lat. Am. J. Aquat. Mamm.* 135-139 (2006).
29. Clua, E. E., Manire, C. A., & Garrigue, C. Biological data of pygmy killer whale (*Feresa attenuata*) from a mass stranding in New Caledonia (South Pacific) associated with hurricane Jim in 2006. *Aquat. Mamm.* **40**, 162-172 (2014).
30. Bryden, M. M. Observations on a Pygmy Killer Wale, *Feresa attenuata*, stranded on the East Coast of Australia. *Wildl. Res.* **3**, 21-28 (1976).
31. González, J. A., Martín, L., Herrera, R., González-Lorenzo, G., Espino, F., & Barquín-Diez, J., *et al.* Cirripedia of the Canary Islands: distribution and ecological notes. *J. Mar. Biolog. Assoc. U.K.* **92**, 129-141 (2012).
32. Caldwell, D. K., Caldwell, M. C., Rathjen, W. F., & Sullivan, J. R. Cetaceans from the Lesser Antillean Island of St. Vincent. *Fish. Bull.* **69**, 303-312 (1971).
33. Carvalho, V. L., Bevilaqua, C. M. L., Iñiguez, A. M., Mathews-Cascond, H., Bezerra Ribeiro, F., Bezerra Pessoae, L. M., *et al.* Metazoan parasites of cetaceans off the northeastern coast of Brazil. *Vet. Parasitol.* **173**, 116-122 (2010).
34. Batista, R. L. G., Schiavetti, A., Santos, U. A. D., & Reis, M. D. S. S. D. Cetaceans registered on the coast of Ilhéus (Bahia), northeastern Brazil. *Biota Neotropica* **12**, 31-38 (2012).
35. Balbuena, J. A., & Raga, J. A. Ecology and host relationships of the whale-louse *Isocyamus delphini* (Amphipoda: Cyamidae) parasitizing long-finned pilot whales (*Globicephala melas*) off the Faroe Islands (Northeast Atlantic). *Can. J. Zool.* **69**, 141-145 (1991).
36. Raga, J. A., & Balbuena, J. A. Parasites of the long-finned pilot whale, *Globicephala melas* (Traill, 1809), in European waters. *Rep. Int. Whal. Commn.* **14**, 391-406 (1993).
37. Hoek, P. P. C. Report on the Cirripedia collected by H.M.S. Challenger during the years 1873–1876. Report of the Scientific Results from the Exploratory Voyages of H.M.S. Challenger. *Zoology* **8**, 1-169 (1883).
38. Nilsson-Cantell, C. A. Cirripeden-Studien. Zur Kenntnis der Biologie, Anatomie und Systematik dieser Gruppe. *Zoologiska Bidrag Fra ̊n Uppsala* **7**, 395 (1921).
39. Nilsson-Cantell, C. A. Cirripedia Thoracica and Acrothoracica. Marine Invertebrates of Scandinavia, *Universitetsforlaget* **5**, 1-133 (1978).

40. Rappé G., & Van Waerebeek, K. "*Xenobalanus globicipitis* (Crustacea: Cirripedia) on cetaceans in the northeast Atlantic and the Mediterranean: a review" in European research on cetaceans, ed. P. G. H. Evans (Lisboa: European Cetacean Society), 75-78 (1988).
41. Steenstrup, J. J. S. Om *Xenobalanus globicipitis*, en ny Cirriped-Slaegt af Coronula familien. *Vidensk Medd Dan Naturh Foren* yr 62-64 (1851).
42. Weltner, W. Verzeichnis der bisher beschriebene recenten Cirripedienarten. Mit Angabe der im Berliner Museum vorhandenen species und ihrer Fundorte. *Archiv für Naturgeschichte* **63**, 227-280 (1897).
43. Gruvel, J. A. Mission Gruvel sur la côte occidentale d'Afrique (1909– 1910) et collection du Museum d'Histoire Naturelle. Les Cirripedes. Paris: *Bull. Mus. Natl. Hist. Nat.* **18**, 344-350 (1912).
44. Zullo, V. A. A preliminary report on the systematics and distribution of barnacles (Cirripedia) of the Cape Cod region. Systematics and Ecology Program, Marine Biological Laboratory, Woods Hole, Massachusetts Contribution **3**, 1-33 (1963).
45. Raga, J. A., Raduan, A., Blanco, C. (1983). Sobre la presencia de *Isocyamus delphinii* (Guerin-Meneville, 1836) (Amphipoda: Cyamidae) en aguas del Mediterráneo español. Actas del I Congreso Ibérico de Entomologia, Facultad de Biología, León 1(2), 627-630.
46. Gruvel, J. A. "Cirrhipedes provenant des campagnes scientifiques de S.A.S. le Prince de Monaco (1885– 1913)" in Résultas des Campagnes Scientifiques accomplies sur son yacht par Albert ler (Monaco: Prince Souverain de Monaco), 1-88 (1920).
47. Pilleri, G., & Knuckey, J., Behaviour patterns of some Delphinidae observed in the Western Mediterranean. *Z. Tierpsychol.* **26**, 48-72 (1969).
48. Pilsbry, H. A. The sessile barnacles (Cirripedia) contained in the collections of the U.S. National Museum; including a monograph of the American species. *Bull. U.S. Natl. Mus.* **93**, 1-366 (1916).
49. Liouville, J. Cétacés de l'Antarctique. Paris: Deuxième Expédition Antarctique Française (1908-1910) (1913).
50. Cornaglia, E., Rebora, L., Gili, C., & Di Guardo, G. (2000). Histopathological and immunohistochemical studies on cetaceans found stranded on the coast of Italy between 1990 and 1997. J. Vet. Med. Ser. A 47(3), 129-142.
51. Ross, G. J. B. The smaller cetaceans of the south east coast of southern Africa. *Ann. Cape Prov. Mus. Nat. Hist.* **15**, 173-410 (1984).
52. Mcalpine, D. F., Murison, L. D., & Hoberg, E. P. New records for the pygmy sperm whale, *Kogia breviceps* (Physeteridae) from Atlantic Canada with notes on diet and parasites. *Mar. Mamm. Sci.* **13**, 701-704 (1997).
53. Allen, G. M. Pygmy sperm whale in the Atlantic. *Field Mus. Nat. Hist. Zool. Ser.* **27**, 17-36 (1941).
54. Prestridge, H. Biodiversity Research and Teaching Collections - TCWC Marine Invertebrates. https://www.gbif.org/occurrence/1234577280 [Accessed March 2, 2021] (2016).
55. Schick, L., IJsseldijk, L. L., Grilo, M. L., Lakemeyer, J., Lehnert, K., Wohlsein, P., *et al.* Pathological findings in white-beaked dolphins (*Lagenorhynchus albirostris*) and Atlantic white-sided dolphins (*Lagenorhynchus acutus*) from the South-Eastern North Sea. *Front. Vet. Sci.* **7**, 262 (2020).
56. Van Bree, P. J. H., & Smeenk, C. Strandingen van Cetacea op de Nederlandse kust in 1976 en 1977. *Lutra* **20**, 13-18 (1978).
57. Berland, B., Krakstad, J-O., Nöttestad, L., Axelsen, B. E., Vaz-Velho, F., Bauleth- & D'Almeida, G. *Xenobalanus globicipitis* (Crustacea: Cirripedia) on dusky dolphins

(*Lagenorhynchus obscurus*) off Namibia: Hitch-hiker's guide to the seas. 15th Biennial Conference on the Biology of Marine Mammals. Greensboro, NC, U. S. (2003).
58. Best, P. B., & Meÿer, M. A. Neglected but not forgotten—Southern Africa’s Dusky Dolphins in *The Dusky Dolphin* (eds. Würsig, B., & Würsig, M.) 291-311 (Academic Press, 2010).
59. Van Waerebeek, K., Reyes, J. C., & Alfaro, J. Helminth Parasites and Phoronts of Dusky Dolphins Lagenorhynchus Obscurus (Gray 1828) From Peru. *Aquat. Mamm.* **19**, 159-159 (1993).
60. Dans, S. L., Reyes, L. M., Pedraza, S. N., Raga, J. A., & Crespo, E. A. Gastrointestinal helminths of the dusky dolphin, *Lagenorhynchus obscurus* (Gray, 1828), off Patagonia, in the Southwestern Atlantic. *Mar. Mamm. Sci.* **15**, 649-660 (1999).
61. Sergeant, D. E., & Fisher, H. D. The Smaller Cetacea of Eastern Canadian Waters. *J. Fish. Res. Board Can.* **14**, 83-115 (1957).
62. Martín, V., Tejedor, M., Pérez-Gil, M., Dalebout, M. L., Arbelo, M., & Fernández, A. A Sowerby's beaked whale (*Mesoplodon bidens*) stranded in the Canary Islands: the most southern record in the Eastern North Atlantic. *Aquat. Mamm.* **37**, 512-519 (2011).
63. Walker, W. A., & Hanson, M. B. biological observations on stejneger’s beaked whale, *Mesoplodon stejnegeri*, from strandings on Adak Alaska. *Mar. Mamm. Sci.* **15**, 1314-1329 (1999).
64. Mead, J. G. Beaked whales of the genus *Mesoplodon* in *Handbook of marine mammals. Volume 4. River dolphins and the larger toothed whales* (eds. Ridgway, S. H., & Harrison, R.) 349-430 (London, 1989).
65. Pastene, L. A., Numachi, K., Jofre, M., Acevedo, M., & Joyce, G. First record of the Blainville's beaked whale, *Mesoplodon densirostris* Blainville, 1817 (Cetacea: Ziphiidae) in the eastern Pacific. *Mar. Mamm. Sci.* **6**, 82-84 (1990).
66. Debrot, A. O. Notes on a gervais’beaked whale, *Mesoplodon europaeus*, and a dwarf sperm whale, *Kogia simus*, stranded in Curaçao, Netherlands Antilles. *Mar. Mamm. Sci.* **8**, 172-178 (1992).
67. Sekiguchi, K., Klages, N. T. W., & Best, P. B. The diet of strap-toothed whales (*Mesoplodon layardii*). *J. Zool.* **239**, 453-463 (1996).
68. Marlow, B. G. Rare whale washed up on Sydney beach. *Austr. Nat. Hist.* **15**, 164 (1963).
69. Dixon, J. M. A recent stranding of the strap-toothed whale, *Mesoplodon layardi* (Gray) (Ziphiidae) from Victoria, and a review of Australian records of the species. *Vict. Naturalist* **97**, 34-41 (1980).
70. Bordino, P., & González, R. Presencia del parásito *Phyllobothrium* sp. (Cestoda) y del foronte *Conchoderma auritum* (Crestacea, Cirripedia) sobre *Mesoplodon layardii* (Cetacea, Ziphiidae). Buenos Aires, Argentina: V Reunión de Trabajo de Especialistas en Mamíferos Acuáticos de América del Sur (1992).
71. Souza, S. P., Siciliano, S., Cuenca, S., & Sanctis, B. A True’s beaked whale (*Mesoplodon mirus*) on the coast of Brazil: adding a new beaked whale species to the Western Tropical Atlantic and South America. *Lat. Am. J. Aquat. Res.* **4**; 10.5597/lajam00077 (2005).
72. Bachara, W., & Gullan, A. First stranding record of a True’s beaked whale (*Mesoplodon mirus*) in Mozambique. Report WB2016/1 (2016).
73. Savage, K. N., Burek-Huntington, K., Wright, S. K., Bryan, A. L., Sheffield, G., Webber, M., *et al.* Stejneger's beaked whale strandings in Alaska, 1995–2020. *Mar. Mamm. Sci.* **37**, 843-869 (2021).

74. Tajima, Y., Maeda, K., & Yamada, T. K. Pathological findings and probable causes of the death of Stejneger's beaked whales (*Mesoplodon stejnegeri*) stranded in Japan from 1999 and 2011. *J. Vet. Med. Sci.* **13**, 0454; 10.1292/jvms.13-0454 (2015).
75. Porsild, M. P. Scattered observations on narwhals. *J. Mammal.* **41**, 8-13 (1922).
76. Wan, X. L., Zheng, J. S., Li, W. X., Zeng, X. Y., Yang, J. W., Hao, Y. J., & Wang, D. Parasitic infections in the East Asian finless porpoise *Neophocaena asiaeorientalis sunameri* living off the Chinese Yellow/Bohai Sea coast. *Dis. Aquat. Org.* **125**, 63-71 (2017).
77. Kim, M. J., & Sohn, H. Rescue, rehabilitation and release of finless porpoise (*Neophocaena asiaeorientalis*) in Korea. *J. Fish. Mar. Sci. Educ.* **28**, 861-871 (2016).
78. Shiozaki, A., & Amano, M. Population-and growth-related differences in helminthic fauna of finless porpoises (*Neophocaena asiaeorientalis*) in five Japanese populations. *J. Vet. Med. Sci.* **16**, 0421 (2017).
79. Huang, Z., Liu, W., Zheng, C., Li, C., Wang, J., & Jefferson, T. A. Finless porpoises in southern coastal waters of Fujian, China. *Acta Oceanologica Sinica* **22**, 114-119 (2000).
80. Parsons, E. C. M., Overstreet, R. M., & Jefferson, T. A. Parasites from Indo-Pacific hump-backed dolphins (*Sousa chinensis*) and finless porpoises (*Neophocaena phocaenoides*) stranded in Hong Kong. *Vet. Rec.* **148**, 776-780 (2001).
81. Devaraj, M., & Sam Bennett, P. Occurrence of *Xenobalanus globicipitis* (Steenstrup) on the Finless Black Porpoise, *Neomeris phocoenoides* in Indian seas. *India. J. Fish.* **21**, 579-581 (1974).
82. Bossart, G. D., Hansen, L., Goldstein, J. D., Kilpatrick, D. Pathological findings in a rare mass stranding of melon-headed whales (*Peponocephala electra*) in Florida. *Aquat. Mamm.* **33**, 235 (2007).
83. Van Waerebeek, K., Hazevoet, C. J., López Suarez, P., Rodrigues, M. S. D., & Gatt, G. Preliminary findings on the mass stranding of melon-headed whale *Peponocephala electra* on Boavista Island in November 2007, with notes on other cetaceans from the Cape Verde Islands. Technical Report, Fondation Internationale du Banc d'Arguin (2008).
84. Siebert, U., Pawliczka, I., Benke, H., Von Vietinghoff, V., Wolf, P., Pilāts, V., *et al.* Health assessment of harbour porpoises (*Phocoena phocoena*) from Baltic area of Denmark, Germany, Poland and Latvia. *Env. Int.* **143**, 105904 (2020).
85. Siebert, U., Tolley, K., Vikingsson, G. A., Olafsdottir, D., Lehnert, K., Weiss, R., *et al.* Pathological findings in harbour porpoises (*Phocoena phocoena*) from Norwegian and Icelandic waters. *J. Comp. Pathol.* **134**, 134-142 (2006).
86. Reckendorf, A., Everaarts, E., Bunskoek, P., Haulena, M., Springer, A., Lehnert, K., *et al.* Lungworm infections in harbour porpoises (*Phocoena phocoena*) in the German Wadden Sea between 2006 and 2018, and serodiagnostic tests. *Int. J. Parasitol.: Parasites and Wildlife* **14**, 53-61 (2021).
87. Dailey, M., & Stroud, R. Parasites and associated pathology observed in cetaceans stranded along the Oregon Coast. *J. Wildl. Dis.* **14**, 503-511 (1978).
88. Tonay, A. M., & Dede, A. First stranding record of a harbour porpoise (*Phocoena phocoena*) in the Southern Aegean Sea. *Growth* **19** (2013).
89. Bellido, J. J., Castillo, J. J., Farfán, M. Á., Martín, J. J., Mons, J. L., & Real, R. Ejemplar enfermo de marsopa *Phocoena phocoena*. *Galemys* **18**, 1-2 (2006).
90. Vidal, O., Brownell Jr, R. L., & Findley, L. T. Vaquita in *Handbook of marine mammals* (eds. Ridgway, S. H., & Harrison, R. J.) (Academic Press, 1999).
91. Brownell Jr, R. L., Findley, L. T., Vidal, O., Robles, A., & Manzanilla, S. N. External morphology and pigmentation of the vaquita, *Phocoena sinus* (Cetacea: Mammalia). *Mar. Mamm. Sci.* **3**, 22-30 (1987).

92. Reyes, J. C., & Van Waerebeek, K. Aspects of the biology of Burmeister's porpoise from Peru. *Rep. Int. Whal. Commn.* **16**, 349-364 (1995).
93. Nansen, F. Hunting and adventure in the Arctic (London: J. M. Dent & Sons, 1925).
94. Berzin, A. A. The sperm whale. Jerusalem: *Israel Program Sci. Transl.* (1972).
95. Tomilin A. G. Mammals of the SSSR and adjacent countries. Mammals of Eastern Europe and Adjacent Countries. Izdatel'stvo Akademi Nauk SSSR. Jerusalem: *Israel Program Sci. Transl.* [In Russian] (1957).
96. Hamilton, J. E. Report of the committee appointed to investigate biological problems incidental to Belmullet whaling station. *Br. Assoc. Adv. Sci.* 125-161 (1914).
97. Lillie, D. G. Observations on the anatomy and general biology of some members of the larger Cetacea. *Proc. Zool. Soc. Lond.* **3**, 769-792 (1910).
98. O'Connor, B., & Franco, J. M. F. *Conchoderma auritum* (L.) (Cirripedia) recorded from a sperm whale *Physeter catodon* L. washed up at Claddaghduff, Co Galway. *Ir. Nat. J.* **27**, 236-236 (2003).
99. Scheffer, V. B. Organisms collected from whales in the Aleutian Islands. *Murrelet* **20**, 67-69 (1939).
100. Carl, G. C. Personal Communication. British Columbia Provincial Museum (1945).
101. IJsseldijk, L. L., Van Neer, A., Deaville, R., Begeman, L., van de Bildt, M., van den Brand, J. M., *et al.* Beached bachelors: An extensive study on the largest recorded sperm whale *Physeter macrocephalus* mortality event in the North Sea. *PLoS One* **13**, e0201221; 10.1371/journal.pone.0201221 (2018).
102. Oliver, G., & Trilles, J. P. Crustacés parasites et épizoïtes du Cachalot, *Physeter catodon* Linnaeus, 1758 (Cetacea, Odontoceti), dans le Golfe du Lion (Méditerranée occidentale). *Parasite* **7**, 311-321 (2000).
103. Hiro, F. The fauna of Akkeshi Bay. II. Cirripedia. *J. Faculty Sci., Hokkaido University* **4**, 213-229 (1935).
104. Chapman, G., & Santler, J. E. Aspects of the fauna and flora of the Azores. V. Crustacea. *Ann. Mag. Nat. Hist.* **8**, 371-376 (1955).
105. Clarke, R. Sperm whales of the Azores. *Discovery Rep.* 28, 237-298 (1956).
106. Nasu, K. Deformed lower jaw of sperm whale. *Sci. Rep. Whales Res. Inst. Tokyo* **13**, 211-212 (1958).
107. Verrill, A. E. The Bermuda Islands. New Haven, CT. Published by the author (1902).
108. Rice, D. W. Progress report on biological studies of the larger cetacea in the waters off California. *Norsk Hvalfangst-Tid.* **52**, 181-187 (1963).
109. Omura, H. Whales in the Adjacent Waters of Japan. *Sci. Rep. Whales Res. Inst. Tokyo* **4**, 27-113 (1950).
110. Mizue, K. Factory Ship Whaling around Bonin Islands in 1948. *Sci. Rep. Whales Res. Inst. Tokyo* **3**, 106-118 (1950).
111. Engel, M. Encalhe de um cachalote, *Physeter macrocephalus*, provocado por emalhamento em rede de pesca no litoral da Bahia, Brasil. Anais da VI Reunião de Trabalhos de Especialistas em Mamíferos Aquáticos da América do Sul, 24-28 (1994).
112. Matthews, L. H. The sperm whale, *Physeter catodon*. *Discovery Rep.* **17**, 93-168 (1938).
113. Best, P. B. "Social organization in sperm whales, *Physeter macrocephalus*" in Behavior of marine animals, eds. H. E. Winn and B. L. Olla (Boston, MA: Springer), 227-289 (1979).

114. Nilsson-Cantell, C. A. Thoracic cirripedes collected in 1925– 1936. *Discovery Rep.* **18**, 223-238 (1939).
115. Davis, W. M. Nimrod of the Sea; or, the American Whaleman (New York: Harper & Brothers, 1874).
116. Nilsson-Cantell, C. A. Thoracic cirripedes collected in 1925–1927. *Discovery Rep.* **2**, 223–260 (1930).
117. Nilsson-Cantell, C. A. Cirripedien von der Stewart Insel und von Südgeorgien. *Senckenbergiana* **12**, 210-213 (1930).
118. Ohno, M., and Fujino, K. Biological Investigation on the Whales Caught by the Japanese Antarctic Whaling Fleets, Season 1950/51. *Sci. Rep. Whales Res. Inst.* **7**, 125-188 (1952).
119. Cockrill, W. R. Pathology of the cetacea. A veterinary study on whales. *Brit. Vet. J.* **116**, 1-28 (1960).
120. Kakuwa, Z., Kawakami, T., & Iguchi, K. Biological investigation on the whales caught by the Japanese Antarctic whaling fleets in the 1951-52 season. *Sci. Rep. Whales Res. Inst.* **8**, 147-213 (1953).
121. Fabian, H. "Bohr" höhlen an Pottwalzähnen. *Zool. Anz., Ergänzungsband zu Band* **145**, 147-162 (1950).
122. Mizue, K., & Murata, T. Biological investigation on the whales caught by the Japanese Antarctic whaling fleets season 1949–50. *Sci. Rep. Whales Res. Inst. Tokyo* **6**, 73-131 (1951).
123. Newman, W. A., & Ross, A. Antarctic Cirripedia. *Antarctic Res. Ser.* **14**, 1-257 (1971).
124. Slijper, E. J. Whales (London: Hutchinson, 1962).
125. Di Beneditto, A. P. M., & Ramos, R. M. A. Records of the barnacla *Xenobalanus globicipitis* (Steenstrup, 1851) on small cetaceans of Brazil. *Biotemas* **13**, 159-165 (2000).
126. Di Beneditto, A. P. M., & Ramos, R. M. A. Biology and conservation of the franciscana (*Pontoporia blainvillei*) in the north of Rio de Janeiro State, Brazil. *J. Cetacean Res. Manag.* **3**, 185-192 (2001).
127. Soto, J. M. R. Annotated systematic checklist and bibliography of the coastal and oceanic fauna of Brazil. I. Sharks. *Mare Magnum* **1**, 51-120 (2001).
128. Brownell, R. L. Progress report on the biology of the Franciscana dolphin in Uruguayan waters. *J. Fish. Res. Board Can.* **32**, 1073-1078 (1975).
129. Danielewicz, D., Rosas F., Bastida R., Marigo J., Muelbert M., Rodriguez D., *et al.* Report of the working group on biology and ecology. *Lat. Am. J. Aquat. Mamm.* **1**, 25-42 (2002).
130. Pinedo, M. C., Praderi, R., & Brownell, R. L. "Review of the biology and status of the franciscana, *Pontoporia blainvillei*" in Biology and conservation of the river dolphins, eds. W. F. Perrin, R. L. Brownell, K. Zhou, and J. Liu. *Occasional Papers of the International Union for Conservation of Nature Species Survival Commission* **3**, 46-51 (1989).
131. Franco-Trecu, V., Szephegyi, M. N., Doño, F., Forselledo, R., Reyes, F., Passadore, C. *et al.* Marine mammal bycatch by the industrial bottom trawl fishery at the Río de la Plata Estuary and the adjacent Atlantic Ocean. *Lat. Am. J. Aquat. Res.* **47**, 89-101 (2019).
132. Pilleri, G. Behaviour of the *Pseudorca crassidens* (Owen) off the Spanish Mediterranean coasts. *Revue Suisse de Zoologie* **74**, 679-683 (1967).
133. Di Beneditto, A. P. M., & Ramos, R. M. A. Biology of the marine tucuxi dolphin (*Sotalia fluviatilis*) in south-eastern Brazil. *J. Mar. Biolog. Assoc. U.K.* **84**, 1245-1250 (2004).

134. Flach, L., Van Bressem, M. F., Pitombo, F., & Aznar, F. J. Emergence of the epibiotic barnacle *Xenobalanus globicipitis* in Guiana dolphins after a morbillivirus outbreak in Sepetiba Bay, Brazil. *Estuar. Coast. Shelf Sci.* **263**, 107632; 10.1016/j.ecss.2021.107632 (2021).
135. Young, P. S. The superfamily Coronuloidea Leach (Cirripedia, Balanomorpha) from the Brazilian coast, with redescription of Stomatolepas species. *Crustaceana* **61**, 190-212 (1991).
136. Ross, G. J., Heinsohn, G. E., & Cockcroft, V. G. Humpback dolphins *Sousa chinensis* (Osbeck, 1765), *Sousa plumbea* (G. Cuvier, 1829) and *Sousa teuszii* (Kukenthal, 1892) in *Handbook of Marine Mammals* (eds. Ridgway, S. H., & Harrison, R. J.) 23-42 (Academic Press, 1994).
137. Frantzis, A. A long and deep step in range expansion of an alien marine mammal in the Mediterranean: First record of the Indian Ocean humpback dolphin *Sousa plumbea* (G. Cuvier, 1829) in the Greek Seas. *BioInvasions Record* **7** (2018).
138. Lane, E. P., De Wet, M., Thompson, P., Siebert, U., Wohlsein, P., & Plön, S. A systematic health assessment of Indian ocean bottlenose (*Tursiops aduncus*) and Indo-Pacific humpback (*Sousa plumbea*) dolphins incidentally caught in shark nets off the KwaZulu-Natal coast, South Africa. *PLoS One* **9**, e107038; 10.1371/journal.pone.0107038 (2014).
139. Greenwood, A. G., Taylor, D. C., & Gauckler, A. Odontocete parasites-some new host records. *Aquat. Mamm.* **7**, 23-25 (1979).
140. Jefferson, T. A., Odell, D. K., & Prunier, K. T. Notes on the biology of the Clymene dolphin (*Stenella clymene*) in the northern Gulf of Mexico. *Mar. Mamm. Sci.* **11**, 564-573 (1995).
141. Cerioni, S., & Mariniello, L. Metazoi parassiti di *Stenella coeruleoalba* (Cetacea: Delphinidae) spiaggiata lungo le coste laziali dal 1985 al 1991. *Parassitologia* **38**, 505-510 (1996).
142. Resendes, A. R., Juan-Sallés, C., Almeria, S., Majó, N., Domingo, M., & Dubey, J. P. Hepatic Sarcocystosis in a Striped Dolphin (*Stenella coeruleoalba*) From the Spanish Mediterranean Coast. *J. Parasitol.* **88**, 206-209 (2002).
143. Raga, J. A., Carbonell, E., & Raduan, M. A. (1982). Incidencias de parásitos en los cetáceos varados en las costas españolas del Mediterráneo. *Memórias do Museu do Mar. Série Zoológica* **2**, 1-11.
144. Raga, J. A., & Carbonell, E. New dates about parasites on *Stenella coeruleoalba* (Meyen, 1833) (Cetacea: Delphinidae) in the western Mediterranean Sea. *Invest. Cet.* **17**, 207-213 (1985).
145. Aguilar, A., & Raga, J. A. The striped dolphin epizootic in the Mediterranean-Sea. *AMBIO* **22**, 524-528 (1993).
146. Aznar, F. J., Balbuena, J. A., & Raga, J. A. Are epizoites biological indicators of a western Mediterranean striped dolphin die-off? *Dis. Aquat. Org.* **18**, 159-163 (1994).
147. Aznar, F. J., Perdiguero, D., Del Olmo, A. P., Repullés, A., Agustí, C., & Raga, J. A. Changes in epizoic crustacean infestations during cetacean die-offs: the mass mortality of Mediterranean striped dolphins *Stenella coeruleoalba* revisited. *Dis. Aquat. Org.* **67**, 239-247 (2005).
148. Duignan, P. J., Geraci, J. R., Raga, J. A., & Calzada, N. Pathology of morbillivirus infection in striped dolphins (*Stenella coeruleoalba*) from Valencia and Murcia, Spain. *Can. J. Vet. Res.* **56**, 242-248 (1992).

149. Insacco, G., Buscaino, G., Buffa, G., Cavallaro, M., Crisafi, E., Grasso, R., *et al.* Il patrimonio delle raccolte cetologiche museali della Sicilia. *Museologia Scientifica Memorie* **12**, 391-405 [In Italian] (2014).
150. Karaa, S., Insacco, G., Bradai, M. N., & Scaravelli, D. Records of *Xenobalanus globicipitis* on *Balaenoptera physalus* and *Stenella coeruleoalba* in Tunisian and Sicilian waters. *Natura Rerum.* **1**, 55-59 (2011).
151. Oliveira, J. B., Morales, J. A., González-Barrientos, R. C., Hernández-Gamboa, J., & Hernández-Mora, G. Parasites of cetaceans stranded on the Pacific coast of Costa Rica. *Vet. Parasitol.* **182**, 319-328 (2011).
152. Ribeiro, F. B., Carvalho, V. L., Bevilaqua, C. M. L., & Bezerra, L. E. A. First record of *Xenobalanus globicipitis* (Cirripedia: Coronulidae) on *Stenella coeruleoalba* (Cetacea: Delphinidae) in the oligotrophic waters of north-eastern Brazil. *Mar. Biodivers. Rec.* **3**, 1-5 (2010).
153. iNaturalist users, iNaturalist. iNaturalist Research-grade Observations. iNaturalist.org. https://www.gbif.org/occurrence/1880668572 [Accessed May 20, 2021] (2021).
154. Addink, M. J., & Smeenk, C. Opportunistic feeding behaviour of rough-toothed dolphins *Steno bredanensis* off Mauritania. *Zool. Verh.* **334**, 37-48 (2001).
155. Alves-Motta, M. R., Luz-Carvalho, V., Nunes-Pinheiro, D. C. S., Groch, K. R., Gonçalves-Pereira, L., Sánchez-Sarmiento, A. M., *et al.* Facial Squamous Cell Carcinoma and Abdominal Peripheral Nerve Sheath Tumour with Rhabdomyoblastic Differentiation in a Rough-toothed Dolphin (*Steno bredanensis*). *J. Comp. Pathol.* **176**, 122-127 (2020).
156. Barnard, K. H. Contribution to the crustacean fauna of South Africa. No. 7. Cirripedia. *Ann. S. Afr. Mus.* **20**, 1-103 (1924).
157. Natural History Museum Natural History Museum (London) Collection Specimens. https://www.gbif.org/occurrence/1056411821 [Accessed April 2, 2021] (2020).
158. Sakai, M., Hishii, T., Takeda, S., & Kohshima, S. Flipper rubbing behaviors in wild bottlenose dolphins (*Tursiops aduncus*). *Mar. Mamm. Sci.* **22**, 966-978 (2006).
159. Toth, J. L., Hohn, A. A., Able, K. W., & Gorgone, A. M. Defining bottlenose dolphin (*Tursiops truncatus*) stocks based on environmental, physical, and behavioral characteristics. *Mar. Mamm. Sci.* **28**, 461-478 (2012).
160. Toth-Brown, J., & Hohn, A. A. Occurrence of the barnacle, *Xenobalanus globicipitis*, on coastal bottlenose dolphins (*Tursiops truncatus*) in New Jersey. *Crustaceana* **80**, 1271-1279 (2007).
161. Birincioğlu, S. S., Aypak, S., Avcı, H., Birincioğlu, B., İpek, E., & Akkoç, A. N. Pathological and parasitological investigations in an adult bottlenose dolphin (*Tursiops truncatus*). *Kafkas Univ Vet Fak Derg* **23**, 1011-1014 (2017).
162. Dollfus, R. *Xenobalanus globicipitis* Steenstrup (Cirripedia, Thoracica): collected on *Tursiops truncatus* (Montagu) near the northern coast of Morocco. *Bull. Inst. Pêches Marit. Maroc.* **16**, 55-59 [In French] (1968).
163. True, F. W. Observations of the life history of the bottlenose porpoise. *Proc. U. S. Natl. Mus.* **13**, 197-203 (1890).
164. Urian, K. W., Kaufmann, R., Waples, D. M., & Read, A. J. The prevalence of ectoparasitic barnacles discriminates stocks of Atlantic common bottlenose dolphins (*Tursiops truncatus*) at risk of entanglement in coastal gill net fisheries. *Mar. Mamm. Sci.* **35**, 290-299 (2019).
165. Mead, J. G., & Potter, C. W. Natural history of bottlenose dolphins along the central Atlantic coast of the United States in *The bottlenose dolphin* (eds. Leatherwood, S. and Reeves, R.) 165-195 (Academic Press, 1990).

166. Pugliese, M. C., Boettger, S. A., & Fish, F. E. Barnacle bonding: morphology of attachment of *Xenobalanus globicipitis* to its host *Tursiops truncatus*. *J. Morphol.* **273**, 453-459 (2012).
167. Silva, D., Young, R. F., Lavin, A., O'Shea, C., & Murray, E. Abundance and seasonal distribution of the Southern North Carolina estuarine system stock (USA) of common bottlenose dolphins (*Tursiops truncatus*). *J. Cetacean Res. Manag.* **21**, 33-43 (2020).
168. Bearzi, M., & Patonai, K. Occurrence of the barnacle (*Xenobalanus globicipitis*) on coastal and offshore common bottlenose dolphins (*Tursiops truncatus*) in Santa Monica Bay and adjacent areas, California. *Bull. S. Calif. Acad. Sci.* **109**, 37-44 (2010).
169. Fertl, D. Occurrence patterns and behavior of bottlenose dolphins (*Tursiops truncatus*) in the Galveston ship channel, Texas. *Texas J. Sci.* **46**, 299-317 (1994).
170. Watson, A., & Gee, L. E. Laryngeal displacement and asphyxiation by a beheaded sheepshead (*Archosargus probatocephalus*) in a bottlenose dolphin (*Tursiops truncatus*). *Aquat. Mamm.* **31**, 447-452 (2005).
171. Watson, A. G., Stein, L. E., Marshall, C., & Henry, G. A. Polydactyly in a bottlenose dolphin, *Tursiops truncatus*. *Mar. Mamm. Sci.* **10**, 93-100 (1994).
172. García-Godos, I. Captura estacional de cetáceos menores en la caleta de Ancón. Lima: Memoria X Congreso Nacional de Biología (1992).
173. Barros, N. B., & Stolen, M. K. Biology of offshore bottlenose dolphins from east Florida. *Mote Marine Laboratory* (2001).
174. Ronje, E. I., Whitehead, H. R., Piwetz, S., & Mullin, K. D. Field summary for common bottlenose dolphin surveys on the Texas, Gulf of Mexico Ciast, 2014-2016. *Southeast Fisheries Science Center* PRBD-2018-02 (2018).
175. Gittings, S. R., Dennis, G. D., & Harry, H.W. Annotated guide to the barnacles of the northern Gulf of Mexico. *Biol. Oceanogr.* **402**, 1-36 (1986).
176. Karuppiah, S., Subramanian, A., & Obbard, J. P. The barnacle, *Xenobalanus globicipitis* (Cirripedia, Coronulidae), attached to the bottle-nosed dolphin, *Tursiops truncatus* (Mammalia, Cetacea) on the Southeastern coast of India. *Crustaceana* **77**, 879-882 (2004).
177. Rajaguru, A., & Shantha, G. Association between the sessile barnacle *Xenobalanus globicipitis* (Coronulidae) and the bottlenose dolphin *Tursiops truncatus* (Delphinidae) from the Bay of Bengal, India, with a summary of previous records from cetaceans. *Fish. Bull.* **90**, 197-202 (1992).
178. Ueda, K. iNaturalist Research-grade Observations. iNaturalist.org. https://www.gbif.org/occurrence/2563485235 [Accessed June 4, 2021] (2020).
179. Van Waerebeek, K., Waerebeek, K. V., Reyes, J. C., Read, A. J., & McKinnon, J. S. Preliminary Observations of Bottlenose Dolphins From the Pacific Coast of South America in *The Bottlenose Dolphin* (eds. Leatherwood, S. & Reeves, R.) (Academic Press, 1990).
180. Weir, C. R. Cetaceans observed in the coastal waters of Namibe Province, Angola, during summer and winter 2008. *Mar. Biodivers. Rec.* **3** (2010).
181. Gómez-Hernández, I., Serrano, A., Becerril-Gómez, C., Basañez-Muñoz, A., & Naval-Ávila, C. Prevalencia y abundancia relativa de balanos *Xenobalanus globicipitis* presentes en poblaciones de delfín nariz de botella *Tursiops truncatus* en el Golfo de México Sur. *Revista de Biología Marina y Oceanografía* **55**, 172-176 (2020).
182. Azevedo, A., Soares, M. P., de Castro, M. C. T., Lailson-Brito Jr, J., & Gurgel, M. I. Ocorrência de epizoítos em cetáceos na costa do Estado do Rio de Janeiro, Brasil. Resumos do XXI Congresso Brasileiro de Zoologia, Porto Alegre, Brasil, 254 (1996).

183. Orams, M. B., & Schuetze, C. Seasonal and age/size-related occurrence of a barnacle (*Xenobalanus globicipitis*) on bottlenose dolphins (*Tursiops truncatus*). *Mar. Mamm. Sci.* **14**, 186-189 (1998).
184. Díaz-Aguirre, F., Salinas, C., Navarrete, S., Castillo, V., & Castilla, C. First record of the commensal barnacle (*Xenobalanus globicipitis*) on common bottlenose dolphins (*Tursiops truncatus*) in Chile. *Aquat. Mamm.* **38**, 76-80 (2012).
185. Romero, M. A., Fernández, M., Dans, S. L., García, N. A., González, R., & Crespo, E. A. Gastrointestinal parasites of bottlenose dolphins *Tursiops truncatus* from the extreme southwestern Atlantic, with notes on diet composition. *Dis. Aquat. Org.* **108**, 61-70 (2014).
186. Rosso, M., Ballardini, M., Moulins, A., & Würtz, M. Natural markings of Cuvier's beaked whale *Ziphius cavirostris* in the Mediterranean Sea. *Afr. J. Mar. Sci.* **33**, 45-57 (2011).
187. Gauthier, H. Observations sur un Cetacé du genre *Ziphius* mort au large d'Alger. *Bull. Stn. Aquic. Pêche Castiglione* **1**, 181-204 (1938).
188. Bane, G. W., & Zullo, V. A. Observations on a stranded goosebeaked whale (*Ziphius cavirostris*, Cuvier 1823) and its ectocommensal barnacles (*Xenobalanus globicipitis*). *J. Elisha Mitchell Sci. Soc.* **96**, 1-3 (1980).
189. Palacios, D. M., Salazar, S. K., & Day, D. Cetacean remains and strandings in the Galapagos Islands, 1923-2003. *Lat. Am. J. Aquat. Mamm.* 127-150 (2004).
190. Monod, T. H., & Serene, R. Parasitic, commensal, and inquiline crustaceans collected during the Rumphius Expedition II. *Osean. Indones.* **6**, 23-27 (1976).
191. Fordyce, R. E., Mattlin, R. H., & Wilson, G. J. Stranding of a cuvier's beaked whale, *Ziphius cavirostris* Cuvier, 1823, at New Brighton, New Zealand. *Mäuri orä* **7**, 73-82 (1979).
192. Gomerčić, H., Gomerčić, M. D., Gomerčić, T., Lucić, H., Dalebout, M., Galov, A. *et al.* Biological aspects of Cuvier's beaked whales (*Ziphius cavirostris*) recorded in the Croatian part of the Adriatic Sea. *Eur. J. Wildl. Res.* **52**, 182-187 (2006).
193. Araki, J., Kuramochi, T., Machida, M., Nagasawa, K., & Uchida, A. A note on the parasite fauna of the western North Pacific minke whale (*Balaenoptera acutorostrata*). *Rep. Int. Whal. Commn.* **47**, 565-567 (1997).
194. Kuramochi, T., Araki, J., Uchida, Moriyama, N., Takeda, Y., Hayashi, N., *et al.* Summary of parasite and epizoit investigations during JARPN surveys 1994-1999, with reference to stock structure analysis for the western North Pacific minke whales. In IWC Scientific Committee Workshop to Review the Japanese Whaling Programme under Special Permit for North Pacific Minke Whales (JARPN) (2000).
195. Uchida, A., & Araki, J. Ectoparasites and endoparasites in the minke whale (*Balaenoptera acutorostrata*) from the North-Western Pacific Ocean. *J. Japan Vet. Med. Assoc.* **53**, 85-88 (2000).
196. Uchida, A. Prevalence of parasites and histopathology of parasitisation in minke whales (*Balaenoptera acutorostrata*) from the western North Pacific Ocean and the southern sea of Okhotsk. *Rep. Int. Whal. Commn.* **48**, 465-479 (1998).
197. Öztürk, A. A., Dede, A. Tonay, A. M., Danyer, E., & Aytemiz, I. (2015). Stranding of a minke whale on the Eastern Mediterranean coast of Turkey. *J. Black Sea/Mediter. Envir.* **21**, 232-237.
198. Dorsey, E. M., Stern, J. S., Hoelzel, A. R., & Jacobsen, J. Minke whales (*Balaenoptera acutorostrata*) from the west coast of North America: individual recognition and small-scale site fidelity. *Rep. Int. Whal. Commn.* **12**, 357-368 (1990).
199. Towers, J. R., Mcmillan, C. J., Malleson, M., Hildering, J., Ford, J. K. B., & Ellis, G. M. Seasonal movements and ecological markers as evidence for migration of common minke

whales photo-identified in the eastern North Pacific. *J. Cetacean Res. Manag.* **13**, 221-229 (2013).
200. Bertulli C. G., Cecchetti A., Van Bressem M. F., & Van Waerebeek K. Skin disorders in common minke whales and white-beaked dolphins off Iceland, a photographic assessment. *J. Mar. Anim. Ecol.* **5**, 29-40 (2012).
201. Ólafsdóttir, D., & Shinn, A. Epibiotic macrofauna on common minke whales, *Balaenoptera acutorostrata* Lácepede, 1804, in Icelandic waters. *Parasites & Vectors* **6**, 105; 10.1186/1756-3305-6-105 (2013).
202. Best, P. B. Seasonal abundance, feeding, reproduction, age and growth in minke whales off Durban (with incidental observations from the Antarctic). *Rep. Int. Whal. Commn.* **32**, 759-786 (1982).
203. Dailey, M. D., & Vogelbein, W. Parasite fauna of 3 species of Antarctic whales with reference to their use as potential stock indicators. *Fish. Bull.* **89**, 355-365 (1991).
204. Ivashin, M. V. Vneshnie parazity malykh polosatikov Antarktiki. *V. sb. Morskie mlekopitayuschie* 125-127 (1975).
205. Ohsumi, S., Masaki, Y., & Kawamura, A. Stock of the Antarctic minke whale. *Sci. Rep. Whales Res. Inst.* **22**, 75-125 (1970).
206. Sedlak-Weinstein, E. Three new records of cyamids (Amphipoda) from Australian cetaceans. *Crustaceana* **60**, 90-104 (1991).
207. Bushuev, S. G. A study of the population structure of the southern minke whale (*Balaenoptera acutorostrata* Lacepede) based on morphological and ecological variability. *Rep. Int. Whal. Commn.* **40**, 317-324 (1990).
208. Cornwall, I. E. Some North Pacific whale barnacles. *Contrib. Can. Biol. Fish.* **3**, 503-517 (1927).
209. Matthews, L. H. The sei whale *Balaenoptera borealis*. *Discovery Rep.* **17**, 183-290 (1938).
210. Heldt, J. H. Note au sujet de *Xenobalanus globicipitis* Steenstrup sur *Balaenoptera borealis* Lesson en Méditerranée. *Bulletin de la Société d'Histoire Naturelle Tunisie* **3**, 25-28 (1950).
211. Allen, G. M. The Whalebone Whales of New England. *Mem. Boston Soc. Nat. Hist.* **8** (2), 107-322 (1916).
212. Cornwall, I. E. Collecting at Cachalot whaling station. *Can. Field-Nat.* **42**, 9-12 (1928).
213. Rice, D. Synopsis of biological data on the sei whale and Bryde's whale in the eastern North Pacific. *Rep. Int. Whal. Commn.* **1**, 333-336 (1977).
214. Collet, R. On the external characters of Rudolphi's rorqual (*Balaenoptera borealis*). *Proc. Zool. Soc. Lond.* **17-18**, 243-265 (1986).
215. Broch, H. Cirripedia Thoracica von Norwegen und dem norwegischen Nordmeere. Eine systematische und biologisch-tiergeographische Studie. *Skr. Vidensk. Selsk.* **17** (1924).
216. Kawamura, A. Some consideration on the stock unit of sei whales by the aspect of ectoparasitic organisms on the body. *Bull. Jap. Soc. Fish. Oceanogr.* **14**, 38-43 [In Japanese] (1969).
217. Collet, R. Norges Pattedyr. *Kristiania* (1912).
218. Gruvel, J. A. Expédition Antarctique Française du Pourquoi-Pas dirigée par M. le Dr. J.-B. Charcot (1908-1910). Liste de Cirrhipèdes. *Bull. Mus. Nat. Hist. Nat.* **5**, 292 (1911).
219. Mörch, J. A. On the natural history of whalebone whales. *Proc. Zool. Soc. Lond.* 661-670 (1911).

220. Dalla Rosa, L., & Secchi, E. R. Stranding of a blue whale (*Balaenoptera musculus*) in southern Brazil: 'true'or pygmy. *Rep. Int. Whal. Commn.* **47**, 425-430 (1997).
221. Cornwall, I. E. The barnacles of British Columbia. *Br. Col. Prov. Mus. Dept.* **7**, 5-69 (1955).
222. Rice, D. W. Blue whale in *Marine Mammals of Eastern North Pacific and Arctic Waters* (ed. Haley, D.) 30-35 (Pacific Search Press, 1978).
223. Scharff, R. F. The Whale-Fishery in Ireland. *Ir. Nat. J.* **22**, 145-147 (1913).
224. Gambell, R. A pygmy blue whale at Durban. *Norsk Hvalfangst-Tid.* **53**, 66-68 (1964).
225. Mackintosh, N. A., & Wheeler, J. F. G. Southern blue and fin whales. *Discovery Rep.* **1**, 257-540 (1929).
226. Ichihara, T. The pigmy blue whale, *Balaenoptera musculus brevicauda*, a new subspecies from the Antarctic in *Whales, Dolphins and Porpoises* (ed. Norris, K. S.) 79-113 (University of California Press, 1966).
227. Ichihara, T. Review of pygmy blue whale stock in the Antarctic in *Mammals in the Seas* 211-218 (FAO, 1978).
228. Nishiwaki, M., & Hayashi, K. Biological survey of fin and blue whales taken in the Antarctic season 1947–48 by the Japanese fleet. *Sci. Rep. Whales Res. Inst. Tokyo* **3**, 132-190 (1950).
229. Nishiwaki, M., & Oye, T. Biological Investigation on Blue Whales (*Balaenoptera musculus*) and Fin Whales (*Balaenoptera physalus*) caught by the Japanese Antarctic Whaling Fleets. *Sci. Rep. Whales Res. Inst.* **5**, 91-167 (1951).
230. Cocks, A. H. Additional notes on the fin-whale fishery on the north European coast. *Zoologist* **3**, 134-142 (1885).
231. Sars, G. O. Beskrivelse af en ved Lofoten indbjerget Erhval (*Balaenoptera musculus*)" Forhandlinger i Videnskabs-selskabet i Christiana 280 (1866).
232. Nilsson-Cantell, C. A. Cirripédes. Résultats Scientifiques du voyage aux Indes Orientales Néerlandaise de LL. AA. RR. le Prince et la Princesse Léopold de Belgique. *Mémoires du Musée Royal d'Histoire Naturelle de Belgique* **3**, 1-24 (1930).
233. Gagnon, J., & Torgersen, J. Canadian Museum of Nature Crustacea Collection. https://www.gbif.org/occurrence/1804299388 [Accessed June 20, 2021] (2020).
234. Marcer, F., Marchiori, E., Centelleghe, C., Ajzenberg, D., Gustinelli, A., Meroni, V., *et al.* Parasitological and pathological findings in fin whales *Balaenoptera physalus* stranded along Italian coastlines. *Dis. Aquat. Org.* **133**, 25-37 (2019).
235. Wirtz, P., Araujo, R., & Southward, A. J. Cirripedia of Madeira. *Helgoland Mar. Res.* **60**, 207-212 (2006).
236. Anthony, R., & Calvet, J. Recherches faites sur le cétacé capturé a Cètte, le 6 octobre 1904 – *Balaenoptera Physalus* (Linné). *Bull. Soc. Phil.* (1905).
237. Methion, S., & Díaz López, B. First record of atypical pigmentation pattern in fin whale *Balaenoptera physalus* in the Atlantic Ocean. *Dis. Aquat. Org.* **135**, 121-125 (2019).
238. Raga, J. A., & Sanpera, C. Ectoparásitos y epizoítos de *Balaenoptera physalus* (L., 1758) en aguas atlánticas ibéricas. *Inv. Pesq.* **50**, 489-498 (1986).
239. Calman, W. T. A whale-barnacle of the genus *Xenobalanus* from Antarctic Seas. *Ann. Mag. Nat. Hist.* **6**, 165-166 (1920).
240. Japha, A. Weitere Beitrage zur Kenntnis der Walhaut. *Zool. Jahrbuch Suppl.* **12**, 711-718 (1910).
241. Stephensen, K. Cirripedia (including Rhizocephala). *Zoology of Iceland* **3**, 1-11 (1938).

242. Hinton, M. A. C. Reports on papers left by the late Major G. E. H. Barrett-Hamilton relating to the whales of South Georgia (Crown Agents for the Colonies, 1925).
243. Omura, H., Fujino, K., & Kimura, S. Beaked whale *Berardius bairdii* of Japan with Notes on *Ziphius cavirostris*. *Sci. Rep. Whales Res. Inst. Tokyo* **10**, 89-132 (1955).
244. Rice, D. W., & Wolman, A. A. The life history and ecology of the gray whale (*Eschrichtius robustus*). *Am. Soc. Mammal.* **3** (1971).
245. Scordino, J. J., Gosho, M., Gearin, P. J., Akmajian, A., Calambokidis, J., Wright, N. Individual gray whale use of coastal waters off northwest Washington during the feeding season 1984–2011: Implications for management. *J. Cetacean Res. Manag.* **16**, 57-69 (2017).
246. Murase, M., Tajima, Y., Okamoto, M., Matsuishi, T., Yamada, T. K., & Asakawa, M. An ectoparasite and epizoite from a western gray whale (*Eschrichtius robustus*) stranded on Tomakomai, Hokkaido, Japan. *J. Rakuno Gakuen Univ.* **38**, 149-152 (2014).
247. Findley, L. T., & Vidal, O. Gray whale (*Eschrichtius robustus*) at calving sites in the Gulf of California, México. *J. Cetacean Res. Manag.* **4**, 27-40 (2002).
248. Dailey, M. D., Gulland, F. M., Lowenstine, L. J., Silvagni, P., & Howard, D. Prey, parasites and pathology associated with the mortality of a juvenile gray whale (*Eschrichtius robustus*) stranded along the northern California coast. *Dis. Aquat. Org.* **42**, 111-117 (2000).
249. Roest, A. I. *Kogia Simus* and other cetaceans from San Luis Obispo County, California. *J. Mammal.* **51**, 410-417 (1970).
250. Scammon, C. M. The marine mammals of the northwestem coast of North America (John M. Carmany, 1874).
251. Sullivan, R., & Houck, W. Sightings and Strandings of Cetaceans from Northern California. *J. Mamm.* **60**, 828-833 (1979).
252. Wellington, G. M., & Anderson, S. Surface feeding by a juvenile gray whale, *Eschrichtius robustus*. *Fish. Bull.* **76**, 290-293 (1978).
253. Matthews, L. H. Notes on the southern right whale, *Eubalaena australis*. *Discovery Rep.* **17**, 169-182 (1938).
254. Best, P. B. The presence of coronuline barnacles on a southern right whale *Eubalaena australis*. *S. Afr. J. Mar. Sci.* **11**, 585-587 (1991).
255. Pettis, H. M., Rolland, R. M., Hamilton, P. K., Brault, S., Knowlton, A. R., Kraus, S. D. Visual health assessment of North Atlantic right whales (*Eubalaena glacialis*) using photographs. *Can. J. Zool.* **82**, 8-19 (2004).
256. Omura, H. North Pacific right whale. *Sci. Rep. Whales Res. Inst.* **13**, 1-52 (1958).
257. Ohlin, A. Some remarks on the bottlenose-whale (*Hyperoodon*). *Lunds. Univ. Arskr.* **29**, 1-14 (1893).
258. Smiddy, P. Bottle-nosed whale *Hyperdoon ampullatus* (Forster). *Ir. Nat.' J.* **22**, 165 (1986).
259. Gosse, P. H. Notes on some new or little-known marine animals. *Ann. Mag. Nat. Hist.* **16**, 27-36 (1855).
260. Minton, G., Van Bressem, M. F., Willson, A., Collins, T., Al Harthi, S., Willson, M. S., *et al.* Visual health assessment and evaluation of anthropogenic threats to Arabian Sea humpback whales in Oman. *J. Cetacean Res. Manage.* **23**, 59-79 (2022).
261. Angeletti, S., Cervellini, P. M., & Massola, V. Nuevo registro de ballena jorobada (*Megaptera novaeangliae*) para el Mar Argentino y notas sobre sus epibiontes. *Mastozoología Neotropical* **21**, 319-324 (2014).

262. Paterson, R. A., & Van Dyck, S. Studies of two humpback whales, *Megaptera novaeangliae*, stranded at Fraser Island, Queensland. *Memoirs of the Queensland Museum* **30**, 343-350 (1991).
263. Groch, K. R., Diaz-Delgado, J., Marcondes, M. C., Colosio, A. C., Santos-Neto, E. B., Carvalho, V. L., *et al.* Pathology and causes of death in stranded humpback whales (*Megaptera novaeangliae*) from Brazil. *PloS one* **13**, e0194872; 10.1371/journal.pone.0194872 (2018).
264. Cornwall, I. E. Notes on west American whale barnacles. *Proc. Calif. Acad. Sci.* **13**, 421-431 (1924).
265. Ávila, I. C., Cuellar, L. M., & Cantera, J. R. Crustaceans ectoparasites and epibionts of humpback whales, *Megaptera novaeangliae* (Cetacea: Balaenopteridae), in the Colombian Pacific. *Res. J. Costa Rican Dist. Educ. Univ.* **2**, 177-185 (2011).
266. Jiménez, I. C. A., Reina, L. M. C., & Kintz, J. R. C. Crustáceos ectoparásitos y epibiontes de ballenas jorobadas, *Megaptera novaeangliae* (Cetacea: Balaenopteridae) en el Pacífico Colombiano. *Cuadernos de Investigación UNED* **3**, 177-185 (2011).
267. Osmond, M. G., & Kaufman, G. D. A heavily parasitized humpback whale (*Megaptera novaeangliae*). *Mar. Mamm. Sci.* **14**, 146-149 (1998).
268. Smiddy, P., & Berrow, S. D. Humpback whale *Megaptera novaeangliae* (Borowski). *Ir. Nat.' J.* **24**, 162 (1992).
269. Nishiwaki, M. Humpback whales in Ryukyuan waters. *Sci. Rep. Whales Res. Inst.* **14**, 49-87 (1959).
270. Angot, M. Rapport scientifique sur les expeditions baleinieres autour de Madagascar (saisons 1949 et 1950). *Mém. Inst. Sci. Madagascar, Sér. A* **6**, 439-486 (1951).
271. Fischer, P. Cirrhipèdes de l'Archipel de la Nouvelle-Calédonie. *Bull. Soc. Zool. France* **9**, 355-360 (1884).
272. Borradaile, L. A. Marine Crustaceans. VII. The barnacles in *The fauna and geography of the Maldive and Laccadive Archipelagoes, being an account of the work carried on and of collections made by an expedition during years 1899 and 1900* (ed. Gardiner, J. S.) 440-443 (Cambridge University Press, 1903).
273. Borradaile, L. A. Crustacea. Part III. - Cirripedia. Br. Antarct. Terra Nova Exped. 1910. *Zool.* **3**, 127-136 (1916).
274. Matthews, L. H. The humpback whale, *Megaptera novaeangliae*. *Discovery Rep.* **17**, 7-92 (1937).
275. Dall, W.H. On the parasites of the cetaceans of the N.W. coast of America, with descriptions of new forms. *Proc. Calif. Acad. Sci.* **4**, 299-301 (1872).
276. Guiler, E. R. Supplement to a list of the Crustacea of Tasmania. *Records of the Queen Victoria Museum* **5**, 1-8 (1956).
277. Ivashin, M. V. Obrastaniya i ektoparasity u gorbatykh kitov. *V kn. Morskie mlekopitayuschie*, 80-86 (1965).
278. Symons, H. W., & Weston, R. D. Studies on the humpback whale (*Megaptera nodosa*) in the Bellinghausen Sea. *Norsk Hvalfangsttid.* **47**, 53-81 (1958).
279. Beneden, P. Les cétacés, leurs commensaux et leurs parasites. *Bull. Acad. r. Belg. Cl. Sci.* **29**, 347-368 (1870).
280. Bouvier, E. L. Quelques Arthropodes recueillis aux iles Kerguelen. *Bull. Mus. Nat. d'Hist. Nat.* **2** (1910).
281. Holthuis, L. B., & Fransen, C. H. Interesting records of whale epizoic crustaceans from the Dutch North Sea coast (Cirripedia, Amphipoda). *Nederlandse Faunistische Mededelingen* **21**, 11-16 (2004).

282. Sars, G. O. Fortsatte Bidrag til Kundskaben om vore Bardehvaler. ‚Finnhvalen. og „Knölhvalen. Forhandl Videnskabsselskabs Selskab *Kristiania* **12** (1880).
283. Sars, G. O. An Account of the Crustacea of Norway: Amphipoda. (Vol. 1 and 2) (A. Cammermeyer, 1895).
284. Donnelly, D., McInnes, J. D., Morrice, M., & Andrews, C. Killer whales of Eastern Australia. Killer Whales Australia (2018).
285. Groch, K., Jerdy, H., Marcondes, M., Barbosa, L., Ramos, H., Pavanelli, L., *et al.* Cetacean Morbillivirus Infection in a Killer Whale (*Orcinus orca*) from Brazil. *J. Comp. Pathol.* **181**, 26-32 (2020).
286. Foote, A. D., Vilstrup, J. T., de Stephanis, R., Verborgh, P., Able Nielsen, S. C., Deaville, R., *et al.* Genetic differentiation among North Atlantic killer whale populations. *Mol. Ecol.* **20**, 629-641 (2011).
287. Cortés-Peña D. Orca at Chañaral de aceituno, Freirina, Atacama region, Chile. https://www. facebook.com/groups/CetalFauna/permalink/206670 2686783266. [Accessed May 20, 2021] (2019).
288. Fertl, D., Acevedo-Gutiérrez A., & Darby F. L. A report of killer whales (*Orcinus orca*) feeding on a carcharhinid shark in Costa Rica. *Mar. Mamm. Sci*. **12**, 606-611 (1996).
289. Pitman, R. L., Fearnbach, H., LeDuc, R., Gilpatrick, J. W. J., Ford, J. K. B., & Ballance L.T. Killer whales preying on a blue whale calf on the Costa Rica dome: genetics, morphometrics, vocalisations and composition of the group. *J. Cetacean Res. Manag.* **9**, 151-157 (2007).
290. Denkinger, J., & Alarcon, D. Orcas of the Galápagos Islands. CETACEA Project (2017).
291. Sakai, Y., Hayashi, R., Murata, K., Yamada, T., & Asakawa, M. Records of barnacle, *Xenobalanus globicipitis* Steenstrup, 1851 and whale lice, *Cyamus* sp. from a wild killer whale captured in the Western North Pacific, off Kii Peninsula, Japan. *Japanese J. Zoo Wildl. Med.* **14**, 81-84 (2009).
292. Guerrero-Ruiz, M., & Urbán, J. R. First report of remoras on two killer whales (*Orcinus orca*) in the Gulf of California, Mexico. *Aquat. Mamm.* **26**, 148-150 (2000).
293. Vargas-Bravo, M. H., Elorriaga-Verplancken, F. R., Olivos-Ortiz, A., Morales-Guerrero, B., Lin-Cabello, M. A., & Ortega-Ortiz, C. D. Ecological aspects of killer whales from the Mexican Central Pacific coast: Revealing a new ecotype in the Eastern Tropical Pacific. *Mar. Mamm. Sci.* 1-16 (2020).
294. Richard, J., & Neuville, H. Sur quelques cétacés observés pendant les Campagnes du yacht Princesse Alice. *Memoires de la Societé Zoologique de France* **10**, 100-109 (1897).
295. Visser, I. N., Cooper, T., & Grimm, H. Duration of pseudo-stalked barnacles (*Xenobalanus globicipitis*) on a New Zealand Pelagic ecotype orca (*Orcinus orca*), with comments on cookie cutter shark bite marks (*Isistius* sp.); can they be used as biological tags? *Biod. J.* **11**, 1067-1086; 10.31396/biodiv.jour.2020.11.4.1067.1086 (2020).
296. Pacheco, A. S., Castro, C., Carnero-Hauman, R., Villagra, D., Pinilla, S., Denkinger, J., *et al.* Sightings of an adult male killer whale match humpback whale breeding seasons in both hemispheres in the Eastern Tropical Pacific. *Aquat. Mamm.* **45**, 320-326 (2019).
297. Best P. B. Whales and Dolphins of the Southern African Subregion (Cambridge University Press, 2007).
298. Whitehead, T. O., Rollinson D. P., & Reisinger R. R. Pseudostalked barnacles *Xenobalanus globicipitis* attached to killer whales *Orcinus orca* in South African waters. *Mar. Biodivers. Rec.* **45**, 873-876 (2014).

299. Samaras, W. F. New host record for the barnacle *Cryptolepas rhachianecti* Dall, 1872 (Balanomorpha: Coronulidae). *Mar. Mamm. Sci.* **5**, 84-87 (1989).
300. Matthews, C., Ghazal, M., Lefort, K., & Inuarak, E. Epizoic barnacles on Arctic killer whales indicate residency in warm waters. *Mar. Mamm. Sci.* **36**, 1010-1014 (2020).
